# Chaperones vs. oxidative stress in the pathobiology of ischemic stroke

**DOI:** 10.3389/fnmol.2024.1513084

**Published:** 2024-12-11

**Authors:** Vladislav Soldatov, Artem Venediktov, Andrei Belykh, Gennadii Piavchenko, Mukhammad David Naimzada, Nastasya Ogneva, Natalia Kartashkina, Olga Bushueva

**Affiliations:** ^1^Department of Pharmacology and Clinical Pharmacology, Belgorod State National Research University, Belgorod, Russia; ^2^Department of Human Anatomy and Histology, I.M. Sechenov First Moscow State Medical University (Sechenov University), Moscow, Russia; ^3^Pathophysiology Department, Kursk State Medical University, Kursk, Russia; ^4^Research Institute of General Pathology, Kursk State Medical University, Kursk, Russia; ^5^Research Institute of Experimental Medicine, Kursk State Medical University, Kursk, Russia; ^6^Laboratory of Public Health Indicators Analysis and Health Digitalization, Moscow Institute of Physics and Technology, Dolgoprudny, Russia; ^7^Scientific Center of Biomedical Technologies, Federal Medical and Biological Agency of Russia, Moscow, Russia; ^8^Laboratory of Genomic Research, Research Institute for Genetic and Molecular Epidemiology, Kursk State Medical University, Kursk, Russia; ^9^Department of Biology, Medical Genetics and Ecology, Kursk State Medical University, Kursk, Russia

**Keywords:** chaperones, stroke, HSP, ROS, hero-proteins

## Abstract

As many proteins prioritize functionality over constancy of structure, a proteome is the shortest stave in the Liebig's barrel of cell sustainability. In this regard, both prokaryotes and eukaryotes possess abundant machinery supporting the quality of the proteome in healthy and stressful conditions. This machinery, namely chaperones, assists in folding, refolding, and the utilization of client proteins. The functions of chaperones are especially important for brain cells, which are highly sophisticated in terms of structural and functional organization. Molecular chaperones are known to exert beneficial effects in many brain diseases including one of the most threatening and widespread brain pathologies, ischemic stroke. However, whether and how they exert the antioxidant defense in stroke remains unclear. Herein, we discuss the chaperones shown to fight oxidative stress and the mechanisms of their antioxidant action. In ischemic stroke, during intense production of free radicals, molecular chaperones preserve the proteome by interacting with oxidized proteins, regulating imbalanced mitochondrial function, and directly fighting oxidative stress. For instance, cells recruit Hsp60 and Hsp70 to provide proper folding of newly synthesized proteins—these factors are required for early ischemic response and to refold damaged polypeptides. Additionally, Hsp70 upregulates some dedicated antioxidant pathways such as FOXO3 signaling. Small HSPs decrease oxidative stress via attenuation of mitochondrial function through their involvement in the regulation of Nrf- (Hsp22), Akt and Hippo (Hsp27) signaling pathways as well as mitophagy (Hsp27, Hsp22). A similar function has also been proposed for the Sigma-1 receptor, contributing to the regulation of mitochondrial function. Some chaperones can prevent excessive formation of reactive oxygen species whereas Hsp90 is suggested to be responsible for pro-oxidant effects in ischemic stroke. Finally, heat-resistant obscure proteins (Hero) are able to shield client proteins, thus preventing their possible over oxidation.

## 1 Introduction

The global burden inflicted by ischemic stroke is dramatic: the annual number of new cases [7,630,803 in 2019 according to the World Stroke Organization (Feigin et al., 2022)] is close to the population of Hong Kong. Similarly, the number of living people who have had at least one ischemic stroke for the mentioned year [77,192,498 (Feigin et al., 2022)] approaches the population of Germany.

Since an acute occlusion of large blood vessels is an unlikely event before the end of the peak of the reproductive period (Putaala et al., 2009), natural selection is mostly “blind” to ischemic stroke and organisms have yet to evolve reliable strategies to fight this disease. However, brain cells recruit essential evolutionarily conservative mechanisms in ischemia to respond to a severe shortage of oxygen (Leu et al., 2019). First, these mechanisms imply a quick switch to anaerobic pathways of ATP production and a decrease in energy demand. At the same time, the cell engages systems preserving structural integrity threatened by the accumulation of H^+^, Ca^2+^, and excessive production of free radicals (Lee et al., 2000).

Since the proteome is the key driver of every cellular process (including cell repair), one of the primary tasks during ischemia is to maintain the integrity of synthesized proteins as far as possible. Molecular assistants contributing to maintaining the integrity of proteins both in physiological and pathological conditions are called chaperones. According to current knowledge, the chaperome, a general term to define the unique steady-state composition of chaperones and their regulators in a given cell type (Wang et al., 2006b), comprises more than 300 proteins (Brehme et al., 2014), forming several rich families and superfamilies according to some of their members' features and functions. The most studied of them, heat shock proteins (HSPs), have a long history from their discovery in the 1960s to the first clinical trials of HSP-regulating drugs in the early 2010s (Porter et al., 2010) and further (Venediktov et al., 2023).

Although frequently discussed as such, many chaperones are not classified as HSPs. For instance, calnexin (Hammond et al., 1994) or the recently discovered family of Hero proteins (Tsuboyama et al., 2020) do not belong to HSPs. Moreover, classically, chaperones are considered to provide homeostasis of the proteome exclusively. However, some authors use the term “lipid chaperones” to refer to molecules selectively assisting in lipid trafficking (Furuhashi et al., 2011). Similarly, a group of metallochaperones exists facilitating intracellular transport of metal ions to other proteins (Rosenzweig, 2002).

However, by virtue of an immense diversity of various molecules called chaperones we will focus only on the “original” chaperones: those that have multiple protein substrates (Soti et al., 2005). In this review, we summarize their roles in the inner defense of the proteome against oxidative stress during ischemic stroke, focusing on the folding and refolding of substrates and the regulation of free radical production. We also outline the rationale, approaches and difficulties of chaperone-based therapy of ischemic stroke, considering their benefit-risk ratio and efficiency in reperfusion phase.

## 2 Outline of cellular damage during ischemic stroke

A hypoxic cell produces excessive amounts of lactate, which decreases pH. In order to decrease acidosis, a cell facilitates Na^+^/H^+^ exchange resulting in Na^+^-overload. According to the coupled exchanger theory (Allen and Xiao, 2003), excessive levels of Na^+^, in turn, decrease the driving force for Ca^2+^ efflux (O'Donnell and Bickler, 1994; Maulik et al., 2002). The latter is promoted by acute deficiency of nucleoside triphosphates impairing the continuous functioning of ion pumps, which regulate Ca^2+^ levels. The imbalanced concentration of cations causes neuronal depolarization and the release of glutamate, which cannot be taken up by hypoxic astrocytes and further aggravates Ca^2+^ influx. Ca^2+^ is known to stimulate the tricarboxylic acid (TCA) cycle and oxidative phosphorylation (McCormack and Denton, 1993; Groten and MacVicar, 2022). Accumulation of Ca^2+^ provokes cell damage by several mechanisms including activation of proteases and mitochondrial impairment with enhanced metabolic flux and the opening of mitochondrial permeability transition pore (mPTP) (O-Uchi et al., 2014; Tajeddine, 2016).

Although it may seem paradoxical and there have been long-lasting debates about it, hypoxia stimulates a dramatic increase of reactive oxygen species (ROS) production (Hernansanz-Agustín et al., 2014). Under physiological conditions, as much as 0.2%−2% of the electrons leak out of the ETC and interact with oxygen to produce superoxide (${\text{O}}_{2}^{•-}$) or hydrogen peroxide (H_2_O_2_) (Turrens, 2003; Cadenas and Davies, 2000; Zhao et al., 2019). This leakage gives rise to some basic level of ROS which is considered to play an important role in healthy cell signaling (Cobley et al., 2018). During acute hypoxia, precise functioning of ETC is imbalanced leading to oxidative stress (Hernansanz-Agustín et al., 2014). A total of 11 sites of ROS generation have been found in mammalian cells (Brand, 2016) but of those complexes I is suggested to be the main source of ROS during acute ischemia (Hernansanz-Agustín et al., 2017).

Thus, Ca^2+^-induced enhancement of TCA and oxidative phosphorylation rate in the absence of O_2_ favors electron leakage from the respiratory chain, generating superoxide and hydrogen peroxide (Semenza, 2011; Yan et al., 2006). Superoxide, in turn, can be dismutated into hydrogen peroxide with further breakdown by catalases and peroxidases or transformation into hydroxyl radicals (•OH). Not only are H_2_O_2_ and •OH highly toxic themselves but they also launch a chain reaction producing lipid peroxides (Gaschler and Stockwell, 2017), hypochlorous acid (HOCl) (Bushueva et al., 2015; Winterbourn et al., 2016), and reactive nitrogen by uncoupling NO synthase (Toma et al., 2021).

Especially important source of ROS in ischemic stroke is the NADPH oxidase (NOX) enzyme family. NOX enzymes are integral to the ETC in the plasma membrane and are widely distributed throughout brain tissue (Zhang et al., 2016). These enzymes generate free radicals by transferring electrons to molecular oxygen, producing a range of secondary reactive species (Vermot et al., 2021). This process plays a crucial role in energy homeostasis and ROS-dependent cellular signaling under both normal and pathological conditions (Brown and Griendling, 2009). In ischemic stroke models, several pharmacological and reverse genetic studies have confirmed that NOX enzymes contribute to the aggravation of the pathology (Zhang et al., 2016).

Additionally, ischemia leads to the increased production of nitric oxide (NO) from endothelial nitric oxide synthase (eNOS), which under normal conditions helps maintain vascular tone and neuroprotection. However, during ischemia, increased NO production can interact with ROS, specifically superoxide, to form peroxynitrite (ONOO^−^), a highly reactive nitrogen species that causes significant oxidative and nitrative damage to proteins, lipids, and DNA.

**Figure 1:**
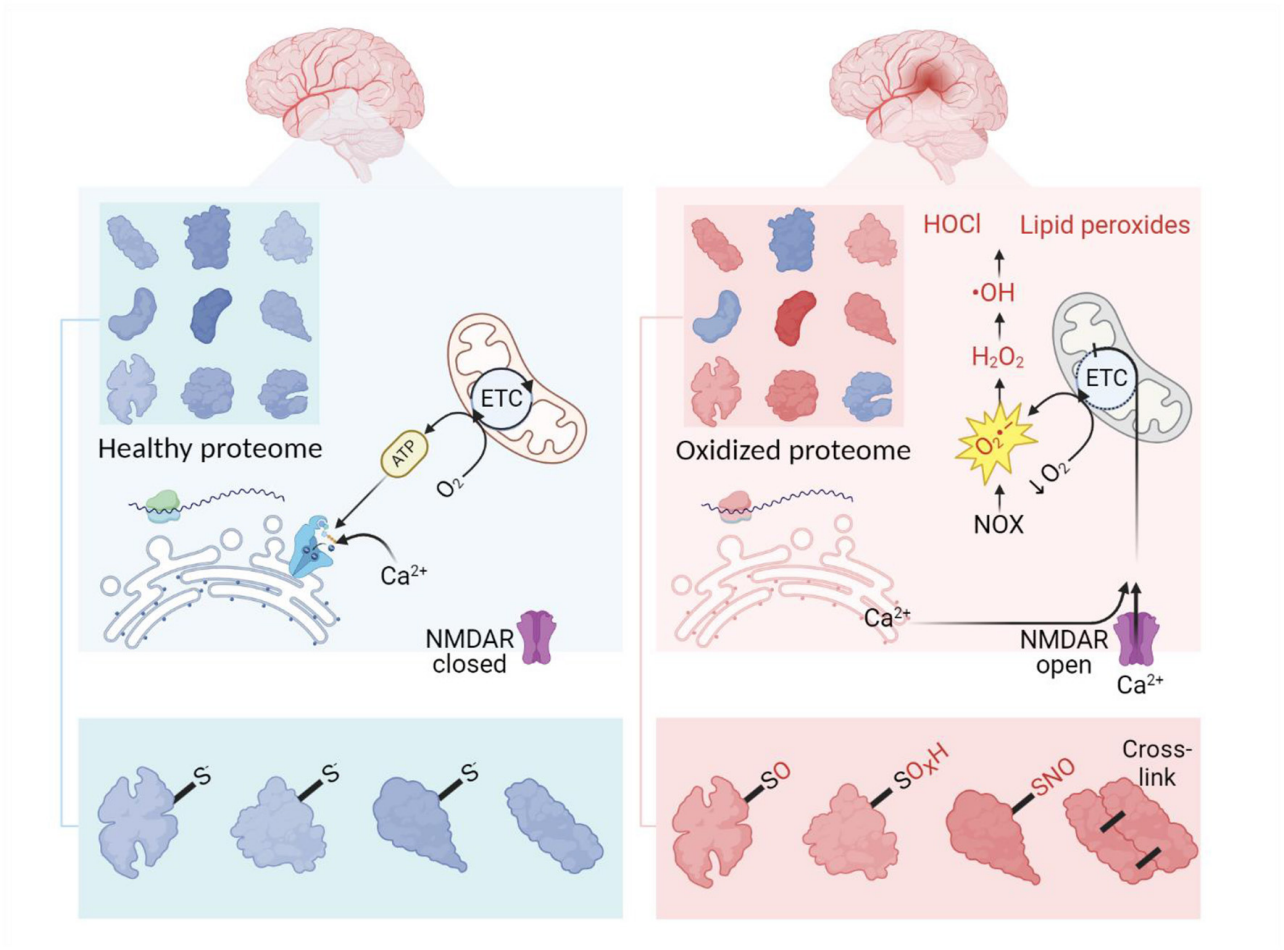
Outlines of oxidative stress and proteome overoxidation in ischemic stroke. Normally, the healthy proteome is supported by balanced translation, modification, and utilization of proteins in homeostatic conditions. At the site of ischemia, a decrease in oxygen provokes mitochondria to release excessive superoxide launching the cascade of ROS production. This process is further enhanced by imbalanced Ca^2+^ as a result of ATP depletion, glutamate excitotoxicity, and increased levels of protons. Developing oxidative stress leads to overoxidation of the proteome, thus complicating the functioning of the proteins. Oxidative modifications of the proteome usually imply the addition of O, OH, O_2_H, O_3_H and NO groups to sulfur-containing amino residues. ETC, electron transport chain. Created in https://BioRender.com.

When overproduced, free radicals inflict oxidative damage to proteins as well as lipids and DNA (Halliwell and Gutteridge, 2015; Reichmann et al., 2018; Caldeira et al., 2014). This oxidative damage causes oxidative modifications of proteins such as cysteine, methionine or tyrosine oxidation, lysine glycoxidation (Kehm et al., 2021), S-nitrosation (Finelli, 2020), self-crosslinking of proteins (Li et al., 2013) (Figure 1), and crosslinking of proteins to DNA (Groehler et al., 2018).

In acute ischemia damaged proteins cannot be recovered by protein turnover and gene expression regulation because the protein translation capacity is limited, the phenomenon called translation arrest (Martín de la Vega et al., 2001). As well as other cell types, facing critical level of proteome oxidation, neurons seek to avoid necrosis inevitably followed by subsequent alteration of neighboring cells (Buttke and Sandstrom, 1994; Reichmann et al., 2018). Hypoxia is known to provoke neuronal apoptosis (Banasiak et al., 2000), ferroptosis (Lan et al., 2020; Yuan et al., 2023; Sanguigno et al., 2023), and autophagy-mediated neuronal death (Shi et al., 2012; Ginet et al., 2014). However, the fact that neurons are implicated in topologically sophisticated circuits and networks makes programmed cell death a barely acceptable scenario. In this regard, one of the most important adaptations is to mobilize antioxidant defenses such as the glutathione system, ROS scavenging (interception of reactive species prior to their reaction with cellular components) (Polonikov et al., 2012, 2022), and antioxidant enzymes (Bushueva et al., 2021; Vialykh et al., 2012), breaking down free radicals.

Cells have evolved several key molecular pathways orchestrating the oxidative stress response. The most important of them rely on Nuclear factor erythroid 2-related factors 1 and 2 (Nrf1 and Nrf2) as well as Forkhead box O (FOXO) transcription factors. While Nrf1 is primarily involved in maintaining cellular homeostasis under basal non-stress conditions (Hu et al., 2022a), Nrf2 is a major regulator of the cellular antioxidant response and activates one of the most important molecular cascades orchestrating antioxidative response in the ischemic brain (Wang et al., 2022b). It induces the expression of a wide range of antioxidant and detoxifying enzymes, such as heme oxygenase-1 (HO-1), glutathione S-transferases, and NAD(P)H oxidoreductase 1 (NQO1) (He et al., 2020). In turn, FOXO transcription factors, such as FOXO3, are also activated under oxidative stress conditions and promote the expression of antioxidant enzymes like superoxide dismutase (SOD) and catalase, DNA repair enzymes, and autophagy-related genes (Morris et al., 2015). This helps to mitigate oxidative damage, repair DNA, and remove damaged proteins or organelles. FOXO3 was shown to exert numerous beneficial effects in stroke (Omorou et al., 2023). Notably, both Nrf2 and FOXO3 are regulated by YAP and TAZ, the components of the Hippo signaling pathway which plays a crucial role in regulating cellular responses to stress, including oxidative stress. It primarily functions to control cell growth, survival, and apoptosis, and its activity can influence how cells respond to oxidative damage (Amanda et al., 2024).

However, the capacity of endogenous antioxidants is limited, and in critical ischemia, a majority of proteins are subjected to chemical stress. At this point cells engage molecular chaperones which exhibit a critical role in protecting proteins from excessive oxidation and aiding in the recovery of damaged proteins during ischemia, thereby helping to preserve cell viability.

## 3 Chaperones in ATP deficiency during ischemic stroke

During ischemic stroke, the loss of blood supply leads to a dramatic reduction in ATP levels. In the ischemic core, ATP is often completely depleted, resulting in inevitable cell death. In the surrounding penumbra, ATP levels are reduced to approximately 30%−50% of normal levels (Liu et al., 2010a; Salaudeen et al., 2024). In these peripheral areas, ATP depletion does not reach critical thresholds, allowing cells to maintain basic functions despite the oxidative stress and other damaging factors they face.

To protect the proteome under low-energy conditions, cells activate chaperones. Although the thermodynamic imbalance worsens under ATP deficiency, cells prioritize the use of available ATP to maintain chaperone function. Chaperones, such as Hsp100, Hsp90, Hsp70, and Hsp60, act as foldases (ATP-dependent enzymes that function according to Michaelis-Menten kinetics) to assist in protein folding and prevent aggregation (Goloubinoff et al., 2018). Foldases are believed to offer a broad range of protective functions, particularly under stress conditions. However, under acutely compromised metabolic states, holdases (ATP-independent chaperones) take over. These holdases, including Hsp27, α-crystallin, and Hero, protect client proteins by non-covalently binding exposed hydrophobic regions, thus preventing improper interactions and aggregation with other proteins (Suss and Reichmann, 2015; Voth and Jakob, 2017; Mitra et al., 2022).

**Figure 2:**
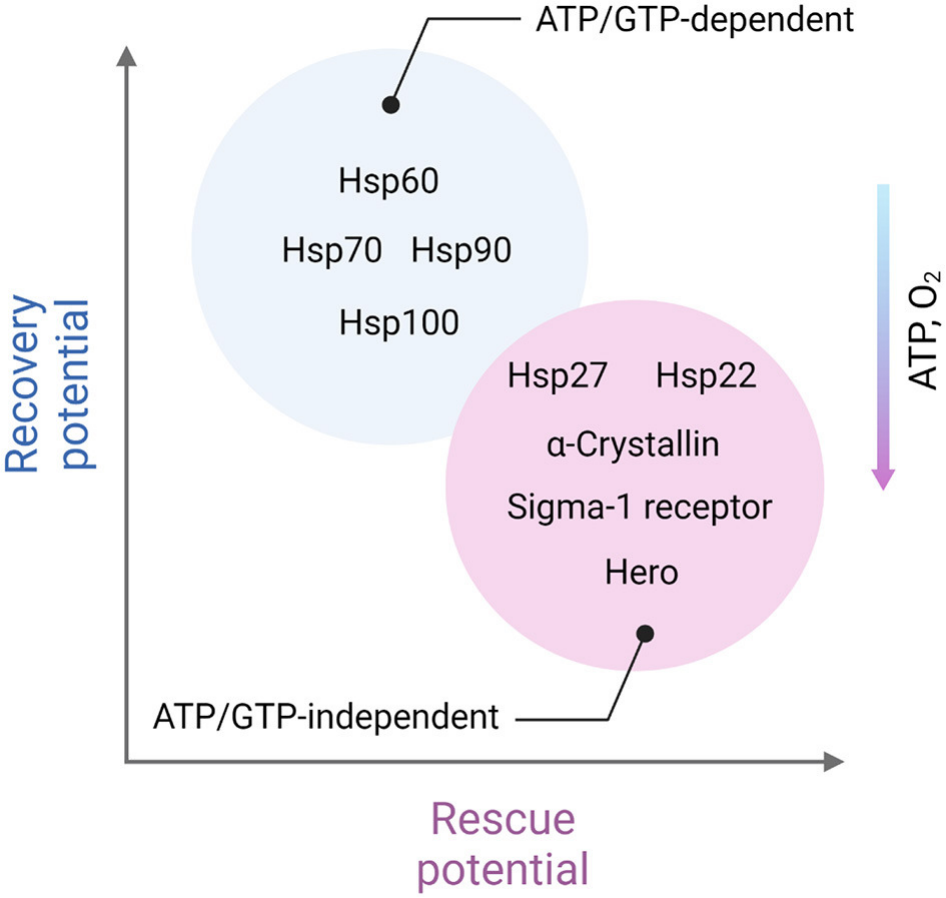
Distinct role of ATP-dependent and ATP-independent chaperones in ischemic stroke. ATP-independent chaperones, including Hsp22, Hsp27, α-crystallin, Sigma-1 receptor, and Hero, are especially vital in areas experiencing severe ATP depletion, closer to the ischemic core and in the acute phase of stroke. These chaperones function without the need for energy input from ATP, making them well-suited to stabilize and protect cellular proteins under energy-deficient conditions. They help prevent protein aggregation by binding to exposed hydrophobic regions of partially unfolded proteins, minimizing cellular damage when energy resources are critically low. On the other hand, ATP-dependent chaperones, which require ATP to assist in protein folding and repair, play a more prominent role in regions with moderate ATP depletion. These chaperones, such as Hsp60, Hsp70, Hsp90, and Hsp100 are particularly important during the recovery phases following the initial stroke event. As ATP levels gradually restore, ATP-dependent chaperones can actively refold and repair damaged proteins, contributing to cellular recovery and enhancing resilience in the affected areas. This complementary activity of ATP-independent and ATP-dependent chaperones reflects a dynamic, phase-specific chaperone response aimed at protecting and restoring the proteome in different stages of stroke-induced cellular stress. Created in https://BioRender.com.

Taking together, one may assume that ATP-independent chaperones such as Hsp27, α-crystallin, and Hero are likely to play a more crucial role in areas of severe ATP depletion and during the acute phase of ischemic stroke. In contrast, ATP-dependent chaperones are essential in regions with less severe ATP loss and are critical for recovery in the later stages of stroke (Figure 2).

## 4 Chaperones against overoxidation

Proteins appear to be significantly more susceptible to oxidative damage in their unfolded states than in their native states, explained by the more open position of side chains (Rollins and Dill, 2014; Zou et al., 2014; Santra et al., 2018; Klyosova and Yu, 2022). Despite the low number of polysomes and many mRNAs being actively translated by just a single ribosome, neurons still have a fairly high translational load, with many mRNAs disseminated throughout different cellular compartments (Biever et al., 2020). Even under the ischemia-induced translation arrest, the cell continues the translation of proteins that constitute the “stress response module” (including chaperones) (Niforou et al., 2014; DeGracia, 2017). For instance, it was shown that nearly half of the detected proteome was altered following stroke. In a murine model of ischemic stroke, affected regions were characterized by dramatically changed proteome patterns, and this response appeared in the early stages of the pathology (Gu et al., 2021). Of those upregulated proteins the chaperones can be detected in the very early stages after the beginning of ischemia (Sun et al., 2015). Moreover, some chaperones may contribute to these reactive changes of the proteome (Guo et al., 2018).

Interestingly, apart from its function in suppressing oxidative damage, many chaperones have been shown to sense overoxidation (Kumar et al., 2021). The latter is due to reversible modifications in redox-sensitive amino acid side chains (e.g. Cys, Met, and His) or metal centers (Kumsta and Jakob, 2009; Ulrich et al., 2021).

During oxidative stress, chaperones also eliminate irreversibly damaged proteins to protect the cell from abnormal polypeptides and allow reassembly of multiprotein complexes (Kiffin et al., 2004). For instance, chaperones are recruited to release oxidized subunits from ribosomes allowing them to be replaced with undamaged (unoxidized) ones (Yang et al., 2023). Oxidative stress stimulates chaperone-mediated autophagy (CMA) by upregulating lysosomal-associated membrane protein 2 (LAMP2A) and heat shock 70 kDa protein 8 (also designated Hsc70)—the CMA effectors—and also makes proteins more easily degradable by the CMA pathway via oxidative modifications (Le et al., 2022; Stricher et al., 2013). CMA is a mechanism known to rescue proteostasis imbalanced by oxidative stress in aging (Zhang and Cuervo, 2008; Schneider et al., 2015). Moreover, it has been reported that via degrading Kelch-like ECH-associated protein 1 (Keap1) CMA activates Nrf2 pathway (Zhang et al., 2004; Zhu et al., 2022b).

Additional important function of CMA is regulation of mitophagy and mitochondrial fragmentation (Nie et al., 2021), thus lowering ROS (Lei et al., 2021). There is another chaperone assisted molecular mechanism of scavenging the damaged proteins, known as Chaperone-assisted selective autophagy (CASA). CASA refers to a broader class of chaperone-mediated pathways that involve the selective targeting of specific protein aggregates or misfolded proteins to autophagosomes for degradation. This involves the classical autophagy machinery. Whereas CMA is primarily dependent on the Hsc70 and its cochaperone Hsp40, CASA involves Hsp70 and Hsp90 (Tedesco et al., 2023).

Mitochondrial chaperones have also been shown to be essential in supporting the stability of electron transport chain components and mitochondrial structure (Herrmann et al., 1994; Bahr et al., 2022; Vishwanathan and D'Silva, 2022; Adriaenssens et al., 2023). During oxidative stress, some chaperones decrease the activity of the complexes II and IV (Sciacovelli et al., 2013; Yoshida et al., 2013; Guzzo et al., 2014) reducing electron leakage and antagonizing the opening of mPTP (Penna et al., 2018).

## 5 Heat shock proteins

HSPs were first discovered in the early 1960s as the factors upregulated in *Drosophila melanogaster* salivary glands after exposure to heat, hence their name. Subsequently, according to their molecular weight, key HSP actors were divided into five major classes: Hsp60, Hsp70, Hsp90, Hsp110, and the small Hsp (Lindquist and Craig, 1988; Macario, 2007). The primary function of HSPs is assistance in the folding, assembling, and addressing of newly synthesized proteins and in refolding (Gupta et al., 2020) or degradation (Fernández-Fernández et al., 2017) of damaged ones.

Different classes of HSPs utilize various strategies to interact with target proteins and regulate their structural changes and assembly. ATP-independent HSPs are holdases or kinetic traps to bind a substrate or an intermediate agent, thus facilitating protein folding and preventing protein aggregation (Mitra et al., 2022). ATP-dependent HSPs, on the other hand, utilize sophisticated dimensional properties—such as the barrel-like Anfinsen cage structure of Hsp60 for sequestered folding of target proteins or modular clamps of Hsp70 and small Hsp—to protect hydrophobic structures in their targets (Bascos and Landry, 2019).

Under normal conditions, HSPs make up an enormous 5%−10% of the total cellular protein content (Hartl et al., 2011). Perhaps unsurprisingly, no single cellular process avoids these master regulators of proteostasis (Tucker et al., 2009; Kashyap et al., 2014). Apart from their reparative machinery, they regulate various molecular pathways of signaling transduction (Silveira et al., 2023). A large body of evidence shows that HSPs are important links contributing to the risk (Xu et al., 2012), course, and outcomes of ischemic stroke (Mohammadi et al., 2018). For example, in the foci of ischemia, Hsp70 is markedly upregulated (Sharp et al., 2000), and an artificial increase in its expression was shown to significantly reduce ischemic damage (Giffard and Yenari, 2004; Demyanenko et al., 2021). Moreover, the autoimmune response against HSPs, found in patients after single or repeated stroke, has been proposed as a link contributing to atherosclerosis plaque formation in cerebral arteries (Banecka-Majkutewicz et al., 2014; Yakovenko et al., 2015). Triggering an immune response against HSPs present on the vascular endothelium may lead to endothelial damage, potentially contributing to the formation of atherosclerotic plaques. This immune response is believed to be induced by cross-reactivity between human and bacterial HSPs following exposure to bacterial infections (Banecka-Majkutewicz et al., 2014).

In their excellent review, Szyller and Bil-Lula (2021) summarize the immense role of HSPs in oxidative and nitrosative stress during ischemia and reperfusion injury mostly in the focus of cardiology (Szyller and Bil-Lula, 2021). Hereinafter we provide a brief description of the main biochemical properties of HSPs (Table 1) and discuss these chaperones with a precise focus on brain ischemic injury.

**Table 1:** Key heat shock proteins (HSPs) in ischemic stroke.

**Family/Member**		**Functions**	**Localization in cell**	**Kinetics**	**References**
Chaperonins/Hsp60		Folding and refolding of polypeptides in mitochondria Posttranslational modifications of polypeptides in cytosol Depends principally on co-chaperonin Hsp10 Activity is inhibited by nitration/hyperacetylation Interacts with mortalin in mitochondria and cytoplasm	Predominantly mitochondria, less in Golgi, peroxisomes, plasma membrane, cytosol	ATP-dependent foldases	Hartl, 1991; Soltys and Gupta, 1997; Wadhwa et al., 2005; Caruso Bavisotto et al., 2020; Androvitsanea et al., 2021
Hsp70	Hsp70/HspA1	Folding of newly synthesized proteins Anti-apoptotic activity in cytosol cascades Hsp40-related proteostasis: Ubiquitin-proteasome axis CMA together with Hsp90 Micro- and macroautophagy Stronger expression in stress reactions Hsp40-dependent and counterpart of Hsp90	Cytosol, lysosomes, plasma membrane, nucleus	ATP-dependent foldases ATP-independent holdases^*^	Flachbartová and Kovacech, 2013; Penna et al., 2018; Bilog et al., 2019; Androvitsanea et al., 2021
	Hsc70	Constitutive member of Hsp70 family Protection of nascent or unfolded proteins Key regulator of physiological autophagy	Nucleus, less in cytosol and lysosomes	Strong ATP- and Hsp40-dependent foldase	Rutledge et al., 2022; Stricher et al., 2013
	Mortalin/mtHsp70	Refolding of ROS-damaged proteins Iron-sulfur cluster synthesis Protein translocation to and from mitochondria Interacts with Hsp60 via N-terminal region	Predominantly mitochondria, less in cytosol,	ATP-dependent foldase	Wadhwa et al., 2005; Esfahanian et al., 2023
Hsp90		Endoplasmic reticulum-located protein folding CMA together with Hsp70 ATP-independent stress signaling Common antagonism with Hsp70	Either cytosolic, endoplasmic reticulum, or mitochondria-related forms, less in nucleus	ATP-dependent foldases ATP-independent holdases^*^	Jackson, 2012; Rutledge et al., 2022; Androvitsanea et al., 2021
Small Hsp/Hsp20 and HspB8		Prevent aggregation of unfolded proteins Recruit Hsp70 to selective autophagy	Nucleus, nucleolus, cytosol, mitochondria, endoplasmic reticulum	ATP-independent holdases	Woehrer et al., 2015; Androvitsanea et al., 2021; Hu et al., 2022b; Tedesco et al., 2023
Hsp27		Protein quality control, referring to proteasome machinery, especially in oxidative stress Holdase with strong anti-apoptotic activity Prevents mitophagy Co-enhances with Hsp70	Cytosol and nucleus, often associated with cytoskeleton	ATP-independent holdase	Stetler et al., 2009; Androvitsanea et al., 2021; Boyd et al., 2023

^*^Some chaperones are not strictly classified as holdases or foldases, possessing both types of activities.

### 5.1 Hsp70

70-kDa heat shock proteins (Hsp70s) subfamily is abundant and ubiquitous molecular chaperones involved in multiple processes such as assistance in the assembly, folding, refolding as well as membrane translocation of client proteins, and control of substrate degradation and activity of various downstream pathways (Mayer and Bukau, 2005; Fernández-Fernández et al., 2017). Hsp70s are ATP-dependent chaperones and require the cooperation with cochaperones and members of other subfamilies of HSPs (e.g., Hsp40, Hsp100, Hsp90) to perform some of the listed functions. The mechanisms by which Hsp70s assist nascent polypeptides folding and damaged/misfolded proteins refolding apparently rely on transient association of their substrate binding domain with short hydrophobic peptide segments within their substrate proteins (Mayer and Bukau, 2005; Xu, 2018). Notably, Hsp70 interacts with a wide variety of client proteins without having exclusive substrates (Murphy, 2013).

The Hsp70 subfamily occupies a central position in cellular proteostasis (Fernández-Fernández et al., 2017) and neuroprotection (Yenari et al., 1999; Bobkova et al., 2014, 2015; Evgen'ev et al., 2017, 2019; Zatsepina et al., 2021; Belenichev et al., 2023; Demyanenko et al., 2024). Some of Hsp70 polymorphisms were described as preventive in ischemic stroke in humans (Kobzeva et al., 2023), probably due to the reduction of thrombosis (Allende et al., 2016). In various rodent models, Hsp70 upregulation has been repeatedly reported to have attenuative effects on stroke (Kim et al., 2018, 2020; Wang et al., 2019; Chi et al., 2014a,b). Moreover, many studies have shown a reduction of oxidative stress to be an important component of its action (Guo et al., 2018; Kesaraju et al., 2014). For example, Hsp70 family members have been suggested to suppress ROS-induced apoptosis. The suppression may be mediated by the inactivation of pro-apoptotic enzymes (Venediktov et al., 2023) or via the downregulation of positive regulator of apoptosis STI1 (da Fonseca et al., 2021; Beraldo et al., 2013).

Plasma exosomes packed with Hsp70 were shown to protect cells against ischemia-reperfusion injury via suppression of ROS and upregulation of SOD (Jiang et al., 2020). Some studies suggest that Hsp70 is able to directly upregulate transcriptional factors mediating antioxidative defense. For example, Guo and colleagues reported that, during stroke, Hsp70 upregulates FOXO3 (Guo et al., 2018). It is known that FOXO3 activation exert numerous beneficial effects in stroke including that FOXO3 signaling pathway activation inhibits oxidative stress-mediated cell death through activation of autophagy (Deng et al., 2023). However, Vinokurov and colleagues have reported that in *in vivo* model of Parkinson's disease induced by the complex 1 uncoupling agent rotenone, despite on protecting neurons and astrocytes against cell death, exogenous Hsp70 upregulated ROS production (Vinokurov et al., 2024). This finding may be related to partial stimulation of the innate immunity response by virtue of use a non-exosome packed form of Hsp70. Even though, the authors reported successful penetration of Hsp70 into the cells and its distribution in mitochondria, one may expect that some fraction of Hsp70 activated the innate immune response cascades (see below in the section *HSPs and Glia*).

**Figure 3:**
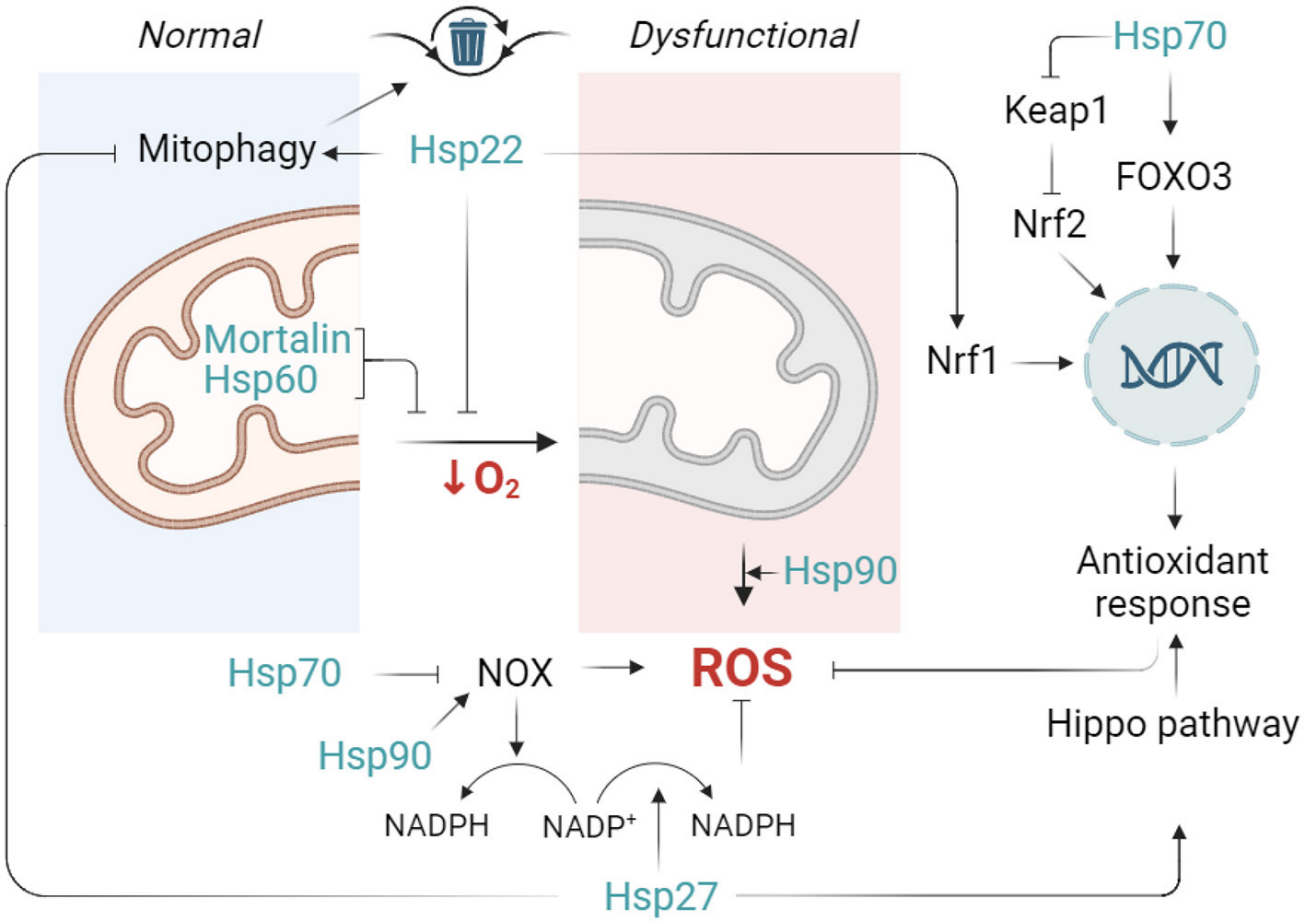
Outlines of the contribution of HSPs to controlling oxidative stress during ischemia. Normal functioning of mitochondria implies moderate leakage of electrons and production of reactive oxygen species (ROS). However, facing a lack of oxygen, mitochondria produce excessive ROS and enter a dysfunctional state. In this scenario, Hsp22 limits mitochondrial activity, thus preventing ROS production. In parallel, mitochondrial Hsp70 (mortalin) and mitochondrial Hsp60 attenuate mitochondrial dysfunction via their chaperone-dependent protection of mitochondrial integrity and ETC. Hsp70 stimulates FOXO3 transcription factor activates Nuclear factor erythroid 2-related factor 2 (Nrf2) pathway and inhibits NOX activity while Hsp22 activates Nuclear factor erythroid 2-related factor 1 (Nrf1). Nrf1 and Nrf2 are both transcription factors known for their roles in regulating the expression of antioxidant and detoxifying enzymes Finally, Hsp27 is able to launch a cellular antioxidant response (such as an increase in superoxide dismutase and glutathione production) through Hippo pathway modulation (and probably other not yet revealed mechanisms), whereas Hsp90 appears to favor ROS production (partly through NOX enzymes activity upregulation). Created in https://BioRender.com.

Some of the Hsp70 family members are integrated in mitochondria and contribute to mitochondrial quality control system. For example, mitochondrial Hsp70 (mortalin) performs two specific roles: as both a chaperone and stress-survival factor, it assists in protein quality control by (re)folding or degrading non-functional proteins as well as in controlling the mitochondrial fragmentation and inflammatory response (Havalová et al., 2021; Zhao et al., 2022) (Figure 3). It has been shown that Mortalin-knockdown murine preadipocyte cells display mitochondrial dysfunction, increased mitochondrial fragmentation, cytosolic mtDNA release and proinflammatory response. Mortalin overexpression increased cell viability, decreased ROS production, preserved mitochondrial membrane potential and ATP levels in primary astrocytes deprived of glucose or oxygen/glucose (Voloboueva et al., 2008). However, Wen et al. have reported that in ischemic brain injury, it was rather the inhibition of mortalin that led to beneficial effects. In their study, inhibition of mortalin was able to effectively ameliorate mitochondrial calcium overload and preserve mitochondrial function in both *in vivo* and *in vitro* stroke models (Wen et al., 2022). Future studies should address the specific roles of mortalin in the regulation of oxidative stress and cells' viability in ischemic stroke.

### 5.2 Hsp90

Hsp90s are five ubiquitous molecular chaperones (HSPC1-5, Kampinga et al., 2009) exerting a wide range of processes which, apart from assistance in protein maturation, degradation, and trafficking, include DNA repair, cell cycle control, cell survival, hormone, and other signaling pathways (Jackson, 2012). Hsp90 has multiple “clients” among which kinases, transcription factors, steroid hormone receptors, and E3 ubiquitin ligases (Schopf et al., 2017). Hsp90 perform their folding activity through a complex process called Hsp90 chaperone cycle where they closely interact with other chaperones. For example, while executing folding, Hsp90 binds the client proteins harbored by the Hsp70/Hsp40 protein complex to start its action (Murphy et al., 2001).

To simplify, in stroke, Hsp90 may be considered to display cellular activity opposite to Hsp70. In this regard, Hsp90 is suggested to exert harmful effects in cerebral ischemic injury (Qi et al., 2015). For example, Hsp90 has been shown to facilitate the pathways of cellular death caused by oxidative damage (Selim and Ratan, 2004) and to activate Acyl-CoA synthetase long-chain family member 4 (ACSL4)—the factor permitting ferroptosis—driven by accumulation of lipid hydroperoxides in ischemic stroke (Miao et al., 2022). In oxidative stress, Hsp90 also promotes the activity of RIP-1 kinase, the main regulator of necroptosis. Notably, the RIP-1-induced cascade additionally stimulates ROS formation, thereby generating a vicious circle of Hsp90-mediated oxidative stress (Zhou et al., 2021). However, another report provided by Wang et al. showed that bardoxolone derivatives drive Hsp90 to perform dephosphorylation of RIP-1 kinase while avoiding necroptosis (Wang et al., 2021a). Noteworthy, in contrast to its stimulative effect on ferroptosis and necroptosis, Hsp90 is a negative regulator of apoptosis. Binding proapoptotic factors Apaf-1, Ask-1, Akt, AIF, and endonuclease G, Hsp90 prevents apoptosis (Sumi and Ghosh, 2022).

In human cerebral microvascular endothelial cells (hCMEC/D3), inhibitors of Hsp90 reduced hydrogen-peroxide-induced ROS generation, suggesting Hsp90 increases free radical production (Uddin et al., 2021). Moreover, Hsp90 up-regulates the functioning of NOX enzymes in ischemic stroke, thereby enhancing generation of ROS (Zhang et al., 2021). For instance, pharmacological and genetic inhibition of Hsp90 directly reduced Nox5-derived superoxide without secondarily affecting downstream signaling events (Chen et al., 2011). Interestingly, Hsp70 and Hsp90 have opposing effects on NOX regulation: while Hsp90 binds to NOX and regulates its stability, these actions are counteracted by Hsp70 (Chen et al., 2012).

Additionally, Hsp90 has been shown to directly interact with Na+/H+ exchanger (NHE)1 (Odunewu-Aderibigbe and Fliegel, 2017a). (NHE)1 is one of the most known triggers of oxidative damage in the cells during hypoxia. Reducing intracellular acidification, it leads to an increase of Na+, followed by activation of Na+/Ca2+ exchanger and subsequent Ca2+ overload. As discussed in Section 2, decompensated increase of Ca2+ leads to oxidative stress. The growing body of evidence shows that NHE1 is deleterious in stroke (Li et al., 2019; Metwally et al., 2023). At the same time, the Hsp90 inhibitor 17-AAG decreased NHE1 activity and NHE1 phosphorylation, suggesting that Hsp90 is a positive regulator of NHE1 (Odunewu-Aderibigbe and Fliegel, 2017b).

Finally, Hsp90 plays a major role in the activation of iNOS and eNOS (Luo et al., 2011; Förstermann and Sessa, 2012), which likely contributes to the aggravation of nitrosative stress in stroke. Taken together, while Hsp90 appears to be a driver of stroke pathology, its anti-apoptotic function and crucial role in orchestrating CASA suggest that it may also exert beneficial effects during stroke recovery.

### 5.3 Hsp60

Hsp60, also referred to as chaperonin 60 (Cpn60), is a foldase typically functioning inside mitochondria together with its co-chaperone Hsp10 to maintain protein homeostasis in ATP- or GTP-dependent manner (Okamoto et al., 2017; Caruso Bavisotto et al., 2020). Apart from its canonical functions in mitochondria, Hsp60 participates in various processes within different cellular compartments being involved in inflammation, cell replication, and other events in health and disease (Macario and Conway de Macario, 2005). For instance, inside mitochondria Hsp60 assists the folding and trafficking of other proteins and prevents mitochondrial protein degradation, but in the cytosol, it can regulate apoptosis (Marino Gammazza et al., 2017; Chaudhuri (Chattopadhyay) and Rashid, 2019).

Hsp60 is considered one of the most important chaperones in sustaining oxidative stress and preserving mitochondrial integrity (Singh et al., 2024). It has been reported to stay upregulated for hours after acute cerebral ischemia (Izaki et al., 2001), but approximately 12–24 h after stroke onset, Hsp60 levels decrease (Kim and Lee, 2007). These temporal dynamics of Hsp60 are suggested to be related to its downregulation by rising iNOS/NO/STAT3, one of the crucial pathways regulating hypoxic response (Kim and Lee, 2007). In hypoxic brain, the role of Hsp60 appears controversial. On the one hand, Hsp60 is beneficial for protein refolding after the harmful impact of ROS. For instance, Cabiscol and colleagues showed that Hsp60 is upregulated in oxidative damage to provide the refolding of iron and sulfur-containing enzymes (Cabiscol et al., 2002). Sheng et al. revealed a negative correlation between the lesion volume and Hsp60 levels in the 1st h after the stroke (Sheng et al., 2011). Importantly, Hsp60 stabilizes respiratory complex I in ETC, and its silencing leads to an increase in ROS levels (Tang et al., 2016). On the other hand, it has been reported that in oxidative stress Hsp60 is responsible for mediating pro-inflammatory signals in astrocytes when it is recognized by innate immune receptors (Liyanagamage and Martinus, 2020). Notably, *in vitro* studies in the serum of ischemic stroke patients have also demonstrated that blocking of Hsp60 prevents inflammatory activation (Brea et al., 2011).

Interestingly, in our previous study, we observed an association between variations in HSPD1, which encodes the Hsp60 family member, and the risk of ischemic stroke, but this association was found exclusively in smokers. This finding further supports the role of Hsp60 in the molecular mechanisms regulating both hypoxia and oxidative stress (Stetskaya et al., 2024).

### 5.4 Hsp27

Hsp27 (HspB1) is constitutively present in cytosol and consists of the N-terminal domain, the α-crystallin domain, and the C-terminal domain. Its chaperone functions do not require ATP but take place due to formation of stable dimers that can further multimerize (Stetler et al., 2009). These multimers provide protein quality control by preventing the formation of protein aggregates, such as for actin filaments, and by directing oxidized proteins along the proteasomal degradation pathway (Arrigo, 2001). In addition, Hsp27 displays pronounced anti-apoptotic activity by downregulation of caspase-3 and caspase-9, the release of cytochrome c, and the inhibition of ASK1 (Stetler et al., 2009; Lanneau et al., 2007). Moreover, Hsp27 has been shown to downregulate the proapoptotic protein Bim binding to the 3′UTR of *bim* mRNA (Dávila et al., 2014).

Hsp27 belongs to chaperones exhibiting eminent neuroprotective activity in various neurological pathologies (Akbar et al., 2003; Shimura et al., 2004; Abisambra et al., 2010) including brain hypoxia and stroke (Stetler et al., 2008; Badin et al., 2006, 2009; Liu et al., 2010b; Tucker et al., 2011; Teramoto et al., 2013; Yu et al., 2013; Leak et al., 2013; Zhan et al., 2017; Shimada et al., 2018; Behdarvandy et al., 2020). Moreover, clinical data indicate marked dynamic changes of Hsp27 levels in patients with acute ischemic stroke (Gruden et al., 2013), suggesting its essential role in hypoxic injury.

Antioxidant role of Hsp27 was repeatedly demonstrated in various models (Vidyasagar et al., 2012; Lin et al., 2016; Önay Uçar et al., 2023). During oxidative stress, Hsp27 was shown to raise the intracellular concentration of the endogenous antioxidant glutathione (Mehlen et al., 1996). Additionally, Hsp27 knockdown reduced the expressions of SOD and catalase (Wang et al., 2022a). Little is known about molecular pathways mediating the upregulation of antioxidant factors by Hsp27. However, some studies suggest the putative role of Hsp27-induced Akt activation (Liu et al., 2007). Moreover, changes in glutathione content may be related to direct interaction between Hsp27 and the enzymes controlling glutathione exchange. Indeed, Lie and coll. demonstrated that Hsp27 overexpression promoted the formation of the complex between Hsp27 and oxidized peroxiredoxin 1 while activating glutathione reductase and thioredoxin reductase in H9c2 cells exposed to hydrogen peroxide (Liu et al., 2019). Additionally, in this study Hsp27 was shown to modulate the Hippo signaling pathway inducing dephosphorylation of MST1 (Liu et al., 2019).

Interestingly, Hsp27 has also been shown to be directly implemented in the regulation of metabolic control of oxidative stress in stroke. In their work on a stroke model in rats Imahori et al. (2017) reported that in hypoxia glucose 6-phosphate dehydrogenase activity in the pentose phosphate pathway (PPP) may be activated via Hsp27 phosphorylation by ATM-kinase. Moreover, pharmacological inactivation of Hsp27 phosphorylation significantly reduced the activity of PPP and resulted in 2-fold increase in infarct size 24-h after reperfusion following 90-min middle cerebral artery occlusion (Yamamoto et al., 2018). Given its essential role in the formation of NADPH, PPP is known as one of the core mechanisms downregulating the oxidative stress under hypoxia (Perl et al., 2011). Thus, direct crosstalk of Hsp27 with PPP indicates its substantial antioxidant role in reducing ischemic damage.

Additionally, one of the putative mechanisms for Hsp27-mediated neuroprotection is the recently discovered role of Hsp27 in suppressing lethal mitophagy by regulation of ceramide synthases and preventing ceramide accumulation in cells (Boyd et al., 2023).

### 5.5 Hsp22

Regardless of the high neuronal expression of small HSPs (Kirbach and Golenhofen, 2011; Bartelt-Kirbach et al., 2017), to date, few studies have described their exact role in the course and outcomes of ischemic brain injury. However, Hsp22 has been shown to directly correlate with the level of mitophagy in mice with middle cerebral artery occlusion and in murine N2A cell cultures subjected to oxygen-glucose deprivation or reoxygenation (Li et al., 2018) as well as in other ischemia models (Cheng et al., 2023). In terms of response to overoxidation, mitophagy is considered an important mechanism contributing to the suppression of oxidative stress (Shao et al., 2020). Increased Hsp22 expression also increased mitochondrial membrane potential and reduced oxidative stress in hippocampal cells of diabetes mellitus mice (Chang et al., 2022).

Hsp22, has been shown to prevent ischemic injury *in vivo* in gerbils and to attenuate oxidative-stress-induced hippocampal neuronal cell death through mitochondrial signaling (Jo et al., 2017). In a rat model of subarachnoid-hemorrhage-induced early brain injury, the exogenous Hsp22 maintained neurological function and reduced brain edema and mitochondrial apoptosis. Furthermore, these effects have been shown to be associated with Nrf1-induced mitochondrial biogenesis and reduction of oxidative stress (Fan et al., 2021).

Thus, the current advance portrays the small HSPs as important players in neuronal injury during stroke. Moreover, the mechanism underlying their contribution appears to be closely related to the regulation of mitochondrial function (Zhu et al., 2015; Fouché et al., 2023). The summarized contribution of HSPs in the regulation of oxidative stress is presented in Figure 3.

### 5.6 HSPs and glial cells

Glial cells, consisting of astrocytes (~20%−40%), microglia (~10%), and oligodendrocytes (~40%−60%), make up about half of the brain's cellular composition (Verkhratsky and Butt, 2018). Given its crucial biological role, glia is considered a dramatically important player in stroke (Jadhav et al., 2023). During the 1st min of stroke glial cells become activated for metabolic cooperation with neurons (Bonvento and Bolaños, 2021), redistribution of cerebral blood flow (Christie et al., 2023), clearance of tissue debris (Jia et al., 2022), and release of neuroprotective molecules (Xie and Liu, 2023), including HSPs (Guzhova et al., 2001; Taylor et al., 2007).

However, glial cells also act as drivers of inflammatory alteration followed by generation of free radicals. A big body of evidence shows that both astrocytes and microglia are rapidly and strongly activated after stroke, generating large amounts of ROS via mitochondrial and NOX pathways. In its turn, free radicals lead to the inflammatory activation of glia exacerbating tissue damage (Zhu et al., 2022a).

Interestingly, HSPs were reported to largely contribute to astrocytes activation in stroke (Barreto et al., 2012). However, heat shock response or Hsp70 alone have been suggested to suppress microglial/brain macrophage activation and astroglia-inducible nitric-oxide synthase expression by decreasing NF-kB activation (Feinstein et al., 1996; Heneka et al., 2000). Align with the last, Hsp70 has been shown to downregulate the expression of pro-inflammatory genes in hypoxia exposed murine astrocytes (Kim et al., 2015). Overexpression of Hsp70 in astrocytes also reduced oxidative stress and rescued glutathione levels in the cells exposed to hydrogen peroxide (Xu and Giffard, 1997).

In contrast, Hsp73 has been suggested to mediate NF-κB and NLRP3 inflammasome activation of astrocytes in ischemic injury (Mi et al., 2021). Similarly, Hsp90 can bind the NLRP3 inflammasome to stabilize its activity (Piippo et al., 2018) leading to NLRP3-induced microglia pyroptosis (Lin et al., 2024). Pyroptosis is a pro-inflammatory form of programmed cell death mostly characteristic for microbial infections (Cookson and Brennan, 2001) but also detected in other conditions including stroke (Chen et al., 2018).

Hsp60 as a ligand for TREM2 receptor on microglial plasma membrane mediates microglial release of pro-inflammatory cytokines, thereby enhancing oxidative stress in neurons (Stefano et al., 2009). Moreover, Hsp60 is required for phosphorylation and nuclear localization of NF-κB after stimulation by the pro-inflammatory cytokine IL-1β (Swaroop et al., 2018).

Notably, the biological effects of HSPs on inflammatory cascades markedly vary depending on their localization: extracellular or intracellular. Being predominantly intracellular molecules, secreted HSPs were suggested to act as damage-associated molecular patterns (DAMPs), “danger signals” activating the innate immune response cascades. Extracellular Hsp70 has been shown to display cytokine regulating activity (chaperokine) stimulating sterile inflammation and exaggerating already existing immune response by engaging TLR2 and TLR4 receptors (Asea et al., 2002; Vabulas et al., 2002a,b; Tsan and Gao, 2004; Hulina et al., 2018).

**Figure 4:**
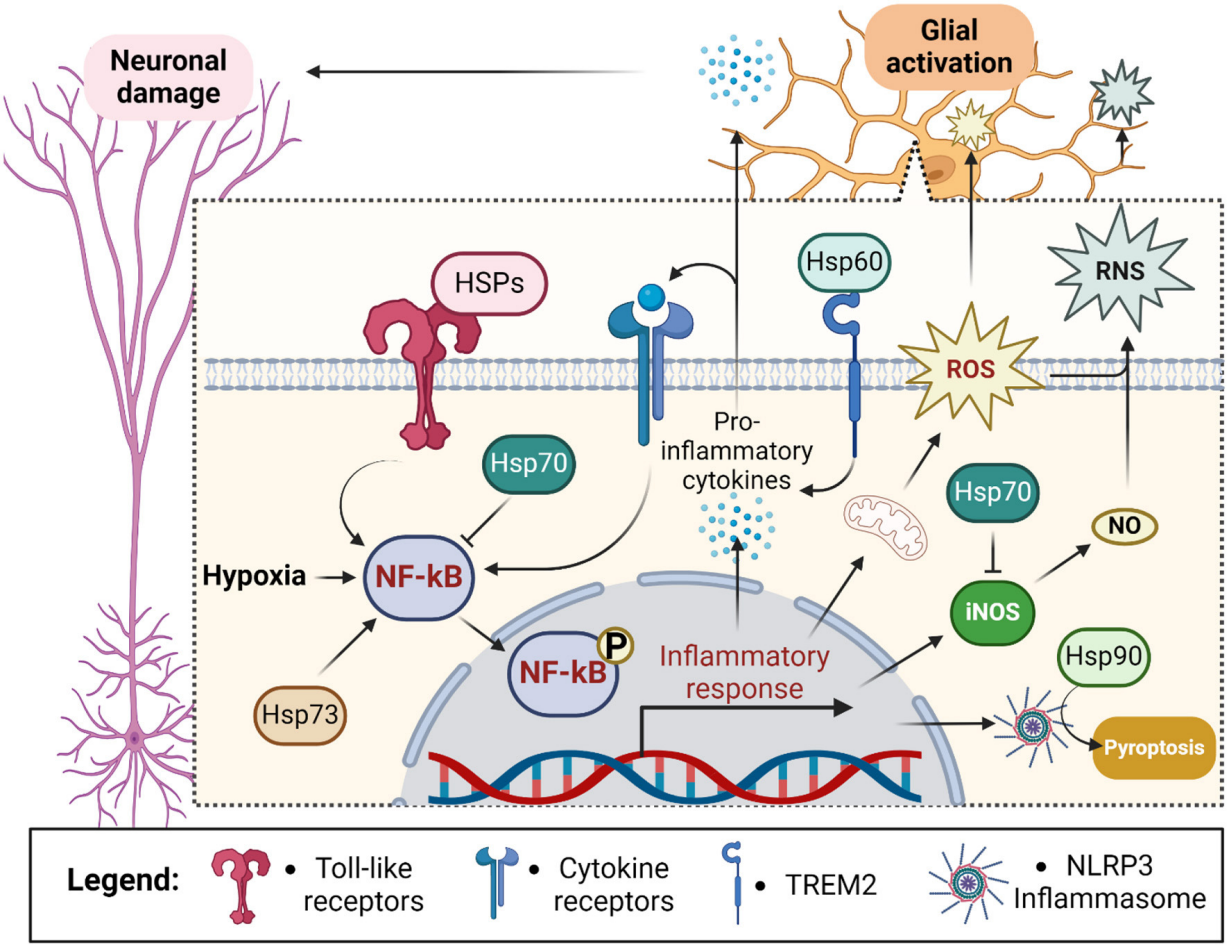
Outline of chaperones' contribution to the glial response during ischemic stroke. Hypoxia stimulates neuroinflammation through the activation of NF-kB in glial cells. NF-kB leads to the assembly of NLRP3 inflammasome and secretion of pro-inflammatory cytokines sending inflammatory stimuli to neurons and glial cells themselves. Glial activation also exacerbates hypoxia-induced reactive oxygen species (ROS) production and leads to the generation of reactive nitrogen species (RNS) via inducible nitric oxide synthase (iNOS)-dependent release of nitric oxide (NO) (Zhu et al., 2022a). Altogether it leads to activation of pyroptosis and damage of proximal neurons. Intracellularly localized Hsp70 prevents activation of NF-kB and iNOS thus suppressing ROS and RNS generation. In contrast, extracellularly localized heat shock proteins (HSPs) activate NF-kB via toll-like receptor signaling and TREM2 (Hsp60). Additionally, intracellular Hsp73 activates NF-kB whereas intracellular Hsp90 stabilizes NLRP3 leading to inflammatory activation and pyroptosis. Created in https://BioRender.com.

Later data have debated their role as DAMPs suggesting HSPs were rather “DAMPERs” (Chen et al., 2011; Chen and Nuñez, 2011; Broere et al., 2011; van Eden et al., 2012), but still, much evidence links extracellular Hsp70 with immune response (Zininga et al., 2018). Due to the antagonistic nature of Hsp70 according to its location, the ratio between extra- and intracellular Hsp70 fractions (Heck index) has been proposed as a marker of inflammatory status (Kim et al., 2015; Krause et al., 2015; Costa-Bebexr et al., 2022; Njemini et al., 2004) (Figure 4).

### 5.7 Hero

In 2020, Tsuboyama and colleagues discovered another family of small chaperones: “Hero proteins.” The authors described six heat-resistant proteins that display strong protective activity against different proteins exposed to damaging factors (Tsuboyama et al., 2020; Morimoto et al., 2022; Tan et al., 2023). Moreover, some of the Hero proteins were shown to decrease the deposition of human TDP-43-positive aggregates both in the eyes of transgenic Drosophila and human induced pluripotent stem (iPS)-derived motor neurons. These proteins were named based on their molecular weights (7, 9, 11, 13, 20, and 45 kDa) as Hero-7, Hero-9, Hero-11, Hero-13, Hero-20, and Hero-45. Although they mostly belong to distinct functional clusters, a common hallmark of these proteins is their remarkable thermostability, hydrophilicity, and disordered structure. Additionally, Hero proteins exhibit unusually high charges: Hero-45, Hero-7, and Hero-11 are highly positively charged, whereas Hero-9, Hero-20, and Hero-13 are highly negatively charged. Their protein-defensive properties are thought to arise from their ability to shield target molecules or function as chaperone holdases, a role facilitated by their flexible structure and unusually high charges. Our group has explored a potential link between Hero proteins and ischemic stroke, revealing that genetic variations in genes encoding Hero protein members are associated with both the risk of ischemic stroke (Kobzeva et al., 2022; Belykh et al., 2023; Shilenok et al., 2023, 2024a,b) and its outcomes (unpublished data).

For instance, we identified that polymorphisms in the Hero- (*C11orf58*) and Hero- (*C19orf53*) genes are significant risk factors for ischemic stroke (IS). Bioinformatics analysis revealed that the SNPs of *C11orf58* and *C19orf53* are implicated in the molecular mechanisms underlying IS, particularly through their involvement in regulating redox homeostasis, inflammation, apoptosis, as well as the response to hypoxia and oxidative stress (Shilenok et al., 2024a,b). Furthermore, bioinformatics analysis using the STRING database (https://string-db.org/) indicated that Hero- and its primary functional partners are associated with six biological processes, primarily related to ATP synthesis. These processes include: ≪mitochondrial respiratory chain complex I assembly≫ (GO:0032981), ≪mitochondrial electron transport, NADH to ubiquinone≫ (GO:0006120); ≪proton motive force-driven mitochondrial ATP synthesis≫ (GO:0042776); proteostasis (≪proteasome-mediated ubiquitin-dependent protein catabolic process≫ (GO:0043161); ≪nucleobase-containing compound biosynthetic process≫ (GO:0034654); and metabolic pathways ≪nitrogen compound metabolic process≫ (GO:0006807) (Shilenok et al., 2024b).

In a separate study, we found that a mutation reducing the expression of Hero-7 (*SERF2*) is also associated with an increased risk of stroke (Belykh et al., 2023). However, there is no conclusive evidence linking SERF2 to the pathobiology of stroke, apart from its involvement in the atherothrombotic component. The neurological function of Hero-7 was examined by Stroo et al. (2023), who demonstrated that mice with brain-specific deletion of SERF2 were viable, exhibited delayed embryonic development, but showed no major behavioral or cognitive abnormalities. These mice were more prone to developing amyloid-beta (Aβ) deposits, and the composition of these deposits was structurally distinct (Stroo et al., 2023). Interestingly, this finding contradicted previous studies, which suggested that SERF1A and SERF2 proteins accelerate the aggregation of multiple amyloidogenic proteins *in vitro* and in C. elegans (Falsone et al., 2012; Yoshimura et al., 2017; Pras et al., 2021; Balasubramaniam et al., 2018). Nevertheless, RNA sequencing data from *Serf2*–/– mice in the study by Stroo et al. (2023) did not reveal significant alterations in cellular pathways typically associated with stroke. Moreover, when we analyzed the available data using bioinformatics tools, there was no strong evidence supporting a role for Hero-7 in oxidative stress, hypoxia response, cell death, or neuroinflammation (Supplementary Table 1).

**Figure 5:**
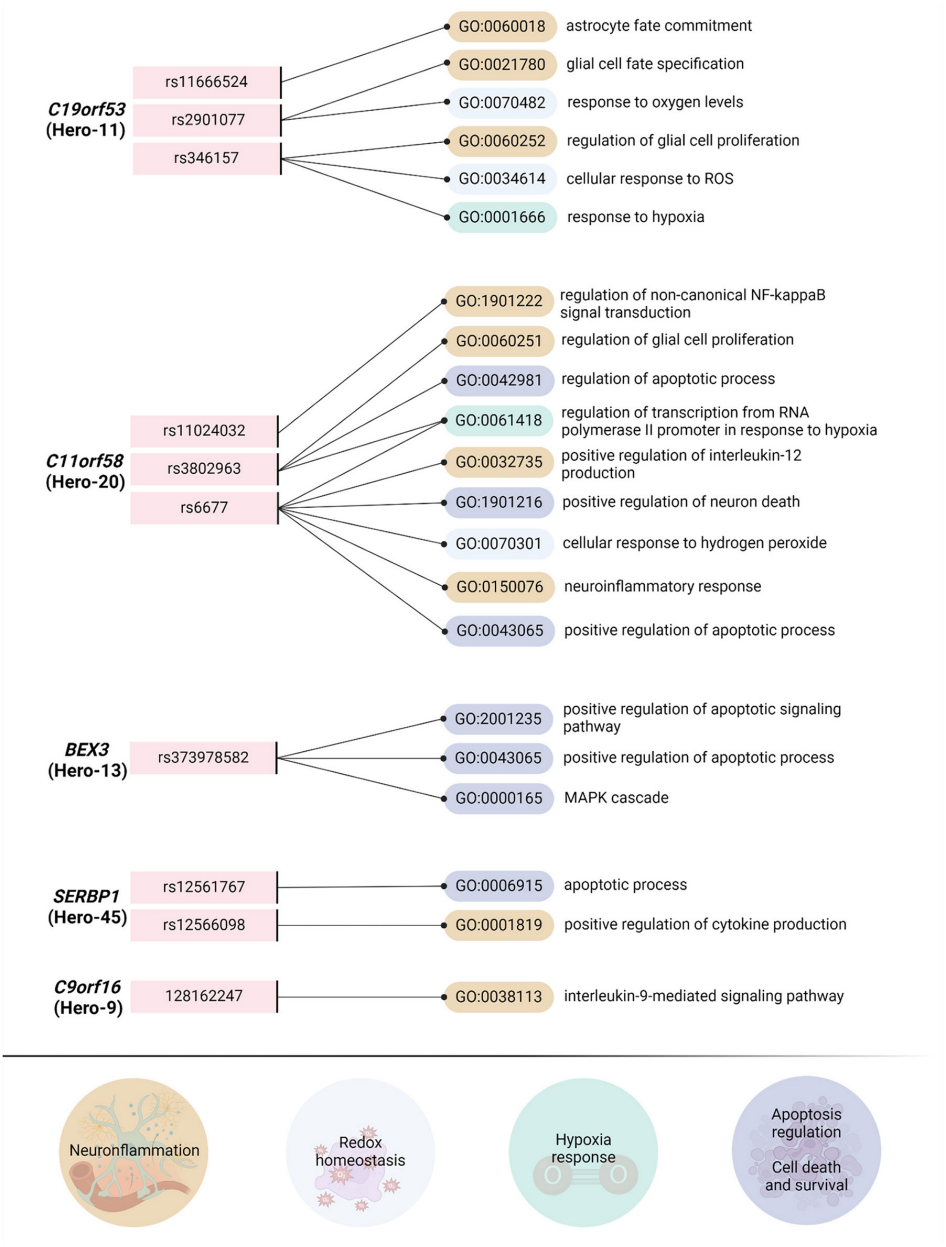
The analysis of overrepresented biological processes based on transcription factors associated with Hero-genetic polymorphisms. Using the Gene Ontology online tool (http://geneontology.org/, accessed on October 23, 2024), we identified overrepresented biological processes directly related to the pathogenesis of ischemic stroke (IS) by analyzing the involvement of transcription factors associated with reference and SNP alleles. For this analysis, we focused on the most frequent single nucleotide polymorphisms (SNPs) or, when available, tagging SNPs (TagSNPs). From the list of biological processes linked to transcription factors associated with the reference and SNP alleles, we selected only those directly relevant to the pathobiology of ischemic stroke. As a result, we classified the biological processes into four groups: “Neuroinflammation,” “Redox Homeostasis,” “Hypoxia Response,” and “Apoptosis Regulation/Cell Death and Survival.” Importantly, we intentionally excluded processes related to neuronal proliferation, migration, and differentiation. The raw data for BEX3, C9orf16, and SERF2 are provided in the Supplementary File. Data for SERBP1, C11orf58 and C19orf53 are presented in Shilenok et al. (2023), Shilenok et al. (2024a), and Shilenok et al. (2024b), respectively. Created in https://BioRender.com.

Another Hero-protein, BEX3, has been identified as a gene with high expression in the brain, particularly in neurons. BEX3 is known to play a role in neuronal apoptosis (Sidhar and Giri, 2017; Figure 5) and neurodegeneration (Park et al., 2000; Yi et al., 2003). Using genetic knockdown animal models, Navas-Pérez et al. (2020) demonstrated that reduced expression of BEX3 results in abnormal brain structures and impaired cognitive function. Furthermore, BEX3 has been shown to directly participate in reactive oxygen species (ROS) sensing in cells. While excessive ROS can induce oxidative stress, their persistent depletion leads to a condition known as reductive stress (Manford et al., 2020). Cells detect reductive stress through the FNIP1 protein (Manford et al., 2020), and BEX3 plays a crucial role in preventing the degradation of FNIP1 (Manford et al., 2021). In their study, Manford et al. (2021) demonstrated that overexpression of BEX3 causes the re-localization of stabilized NRF2 from the nucleus to perinuclear regions. Overall, the high neuronal expression of BEX3, its regulation of apoptosis, and its chaperone function may contribute to its role in ischemic stroke.

Finally, albeit we identified an association between the Hero-45 (*SERBP1*) polymorphism and ischemic stroke, data regarding its role in the response to brain injury are limited. However, its involvement in glioblastoma (Kosti et al., 2020), its presence in stress granules under oxidative stress conditions (Lee et al., 2014), and its strong suppression of TDP-43-induced pathology in the Drosophila eye system (Tsuboyama et al., 2020) suggest that SERBP1 may play a role in cellular responses to brain pathology. Additionally, our bioinformatics analysis revealed that representative single nucleotide polymorphisms (SNPs) of Hero-45 affect the binding of transcription factors involved in apoptosis regulation and cytokine production (Figure 5). This suggests that Hero-45 is involved in these molecular pathways.

### 5.8 Sigma-1 receptor (σ1R)

The Sigma-1 receptor (σ1R) is a molecular chaperone expressed in both neurons and glial cells, primarily localized to the mitochondria-associated endoplasmic reticulum (ER) membranes and plasma membranes (Hayashi and Su, 2005). As a chaperone, σ1R plays a vital role in proteome quality control by facilitating the degradation of misfolded proteins through the ER-associated degradation pathway linked to the ubiquitin-proteasome system (Miki et al., 2014). It is also involved in regulating cellular responses to oxidative stress, where, upon activation, it promotes antioxidant defenses, thereby helping to reduce cellular damage by mitigating oxidative stress (Chu and Ruoho, 2016).

σ1R enhances cellular resilience by interacting with and stabilizing antioxidant proteins, which is especially important under conditions of elevated oxidative stress, such as ischemia. In addition to managing oxidative stress, σ1R contributes to calcium homeostasis and has shown protective effects against both ischemic and non-ischemic brain injury in various animal models (Allahtavakoli and Jarrott, 2011; Wang et al., 2021b; Hong et al., 2015; Orciani et al., 2023; Vagnerova et al., 2006; Ruscher et al., 2011, 2012; Zhang et al., 2017, 2023; Song et al., 2023; Ajmo et al., 2006; Shi et al., 2021). These studies indicate that positive regulation of σ1R helps to attenuate brain damage across a broad spectrum of pathological conditions.

On a molecular level, σ1R enhances antioxidant defenses by interacting with key transcription factors like Nrf2 (Lasbleiz et al., 2022; Li et al., 2024), which regulate the expression of antioxidant proteins, such as SOD, catalase and heme oxygenase-1. σ1R also stabilizes structural proteins, including those associated with the cytoskeleton and cell membranes, helping to maintain cellular integrity under stress conditions. For example, σ1R has been observed to bind with ankyrins and other cytoskeletal components, thereby supporting cell membrane stability and resilience (Hayashi and Su, 2001; Su and Hayashi, 2001).

In the context of ischemic stroke, σ1R plays a critical role in modulating the unfolded protein response (UPR) (Hayashi and Su, 2007; Mori et al., 2013). The UPR is a compensatory mechanism activated to manage the accumulation of misfolded proteins under cellular stress (Hetz, 2012). Enhancing σ1R expression in cells has been shown to mitigate ER stress, while reductions in σ1R levels are associated with increased apoptosis (Hayashi and Su, 2007). Furthermore, σ1R exhibits mitoprotective properties, reduces neuroinflammation, and dampens excitotoxicity, providing further neuroprotection in ischemic conditions (Nguyen et al., 2017; Lu et al., 2012; Wegleiter et al., 2014; Rodríguez-Muñoz et al., 2018).

Interestingly, the role of σ1R in oxidative stress modulation can vary depending on the physiological context. While some studies suggest that σ1R activation promotes antioxidant defenses (Pal et al., 2012), others indicate that the absence of σ1R can also lead to enhanced antioxidant responses. For example, primary neuron-glia cultures obtained from mice with σ1R knockout displayed enhanced Nrf2 antioxidant defense especially when the cells were under stressful conditions (Weng et al., 2017). Goguadze et al. (2019) propose that σ1R may stimulate oxidative stress under normal physiological conditions but provides protection against excessive oxidative damage in pathological states.

**Figure 6:**
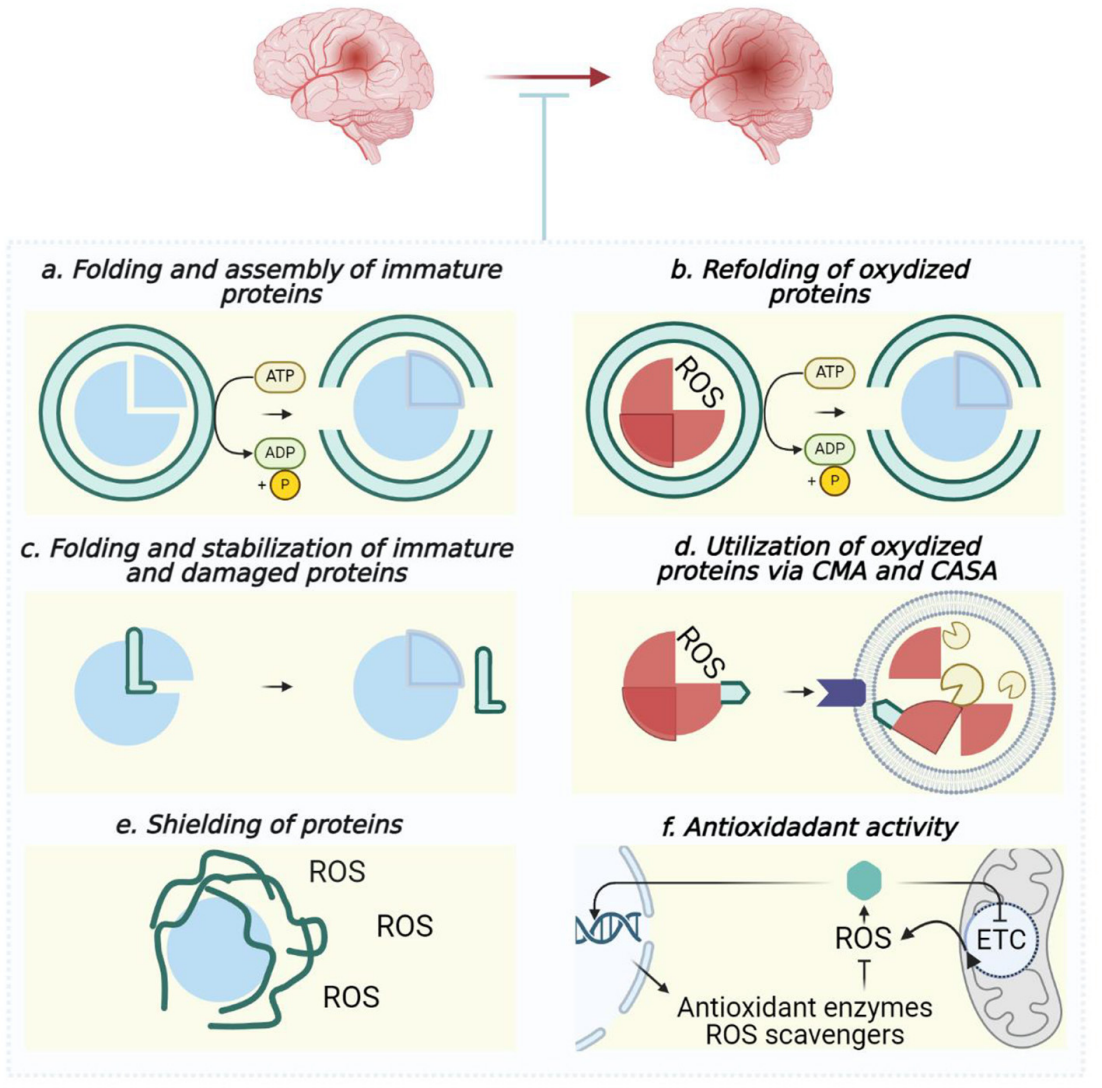
Outline of chaperones' contribution to maintaining proteome quality during ischemic stroke. In normal conditions **(A, C)** and to cope with overoxidation threats **(A–F)**, chaperones co-interact with client proteins as foldases **(A, B)** or holdases **(C)**, facilitate autophagic utilization of damaged polypeptides **(D)**, shield client proteins to scavenge ROS **(E)** as well as provide ROS-sensing and activation of ROS-defense **(F)**. Blue circles, undamaged proteins; red circles, damaged proteins; green objects interacting with blue or red circles, chaperones; round lipid envelope with yellow “pac-mans” inside, lysosome. **(A, B)** Hsp60, Hsp70, Hsp90, and Hsp100; **(C)** Hsp22, Hsp27, α-Crystallin, Sigma-1 receptor, and Hero-proteins; **(D)** Hsc70 (CMA), Hsp70, and Hsp90 (CASA); **(E)** Hero-proteins; **(F)** Hsp22, Hsp27, and Hsp70. Created in https://BioRender.com.

The summarized contribution of σ1R and other chaperones to providing proteome quality control during ischemic stroke is pictured in Figure 6.

## 6 Perspectives

Molecular chaperones are a powerful system maintaining proteome quality control in either healthy or stressed cells. Accordingly, the idea of attenuating neuronal damage via this tool is attractive, although somewhat mechanistic. Despite being mostly anecdotal, myriad pharmacological strategies based on interventions upregulating or delivering various components of chaperone machinery in stroke have already been discussed (Kim et al., 2018; Shao et al., 2019). Nevertheless, many years of research experience and promising results in animal studies of chaperone-based approaches have not yet been successfully translated into clinical practice.

This may reflect that each chaperone is involved in numerous molecular pathways sophisticatedly organized and interconnected together. Thus, any intervention targeting or delivering chaperones is potentially associated to the risk of unbalancing signaling networks.

To date, dozens of clinical trials have investigated heat shock proteins (HSPs), with the majority focusing on cancer therapies, particularly Hsp90 inhibitors such as 17-AAG, XL888, and AT13387. Many trials have also targeted neurodegenerative diseases, aiming to pharmacologically upregulate Hsp70 (see our previous review, Venediktov et al., 2023). For example, an ongoing trial is assessing the efficacy of Arimoclomol in ALS (Benatar et al., 2024). Additionally, some studies have explored the role of HSPs in atherosclerosis (e.g., NCT04787770). The substantial body of clinical research on HSP modulators lays a solid foundation for their potential future application in ischemic stroke. Although dedicated studies on HSPs in stroke patients have not yet been conducted, the existing studies will provide valuable insights into the pharmacodynamics and potential side effects of these modulators.

In principle, as well as other molecules of a protein nature and predominantly cytoplasm-sited activity, chaperones may scarcely be used as a treatment option administered directly. There were successful attempts to utilize chaperones coupled with TAT, the leading sequence from human immunodeficiency virus, or with other cell-penetrating peptides to increase their cellular uptake (Komarova et al., 2015; Hino et al., 2023). This strategy was implemented with some chaperones and showed beneficial effects in models of neuronal pathologies (Wheeler et al., 2003; Lai et al., 2005; Kim et al., 2008), albeit the data were limited by *in vitro* research.

Some small chemicals such as Arimoclomol (Kieran et al., 2004; Cudkowicz et al., 2008; Kalmar et al., 2008), Colchicine (Mandrioli et al., 2019), Phencyclidine (Sharp et al., 1994; Narita et al., 1997), Quercetin (Chander et al., 2014), 17-dimethyl aminoethylamino-17-demethoxygeldanamycin hydrochloride (17-DMAG) (Qi et al., 2014; Zhang et al., 2021; Hu et al., 2023b), and Geldamycin (Xu et al., 2003; Shen et al., 2005; Manaenko et al., 2010, 2011) shown to regulate the activity of various chaperones with beneficial effects in diseases of CNS, can be considered as agents for the intervention in stroke or the post-stroke recovery. However, there is a lack of drugs specifically targeting any chaperone, and the aforementioned molecules are highly unspecific, suggesting that chaperones belong to “undruggable” or “under-drugged” targets (Chan and Tsourkas, 2024). Fortunately, drug design technologies led to the discovery of selective chaperone inhibitors (Ambrose et al., 2023; Hu et al., 2023a) and, hence, may be considered promising candidates to study as ischemic stroke therapeutics.

Effective pharmacological interventions selectively regulating a desired chaperone also might implement gene therapeutic tools, such as lipid nanoparticles, antisense oligonucleotides, siRNA and, especially, adeno-associated viruses. Some previous works have reported successful amelioration of CNS damage (Luecke et al., 2023; Moloney et al., 2014; Fink et al., 1997; Yenari et al., 1998) including ischemia (Chi et al., 2014a,b; Guo et al., 2018; Kesaraju et al., 2014; Badin et al., 2009; Kelly et al., 2002; Hoehn et al., 2001; Wen et al., 2022) via utilization of such approaches. Moreover, recent advances in gene therapeutic approaches enable the precise delivery of molecular constructions to desired organelles, e.g.; to the most important source of ROS: mitochondria (Soldatov et al., 2022). Further focus should obviously be on preclinical testing these approaches in more detail.

Particular interest might be given to the use of chaperones in the reperfusion phase of stroke. Nowadays, interventions restoring the blood flow are considered a golden standard in patients with early ischemic stroke (Hurford et al., 2020). The recanalization may be achieved due to intravenous administration of clog-busting drugs (Wardlaw et al., 2014), endovascular thromboectomy (Goyal et al., 2015), sonothrombolysis (Li et al., 2020) or their combination (Saver et al., 2015; Bracard et al., 2016). However, if a recurrent blood flow takes place, this phase—reperfusion—is known to exacerbate the oxidative stress (Hossmann, 2009; Sun et al., 2018) causing the second phase of alteration. This repeated burst of ROS orchestrates post-reperfusion inflammatory activation, leukocyte-endothelial adhesion, and a breach of the blood-brain barrier (Mandalaneni et al., 2024). Therefore, the treatments which have been proposed to reduce the reperfusion injury mostly imply the antioxidant therapy (Wood and Gladwin, 2007; Wang et al., 2006a).

However, some chaperones exhibit controversial biological effects that vary from positive to negative across various research. Moreover, many chaperones act as a double-edged sword causing different adverse effects, which limits the application of chaperone-based strategies in clinical practice. For instance, since they are secreted extracellularly, HSPs act as damage-associated molecular patterns (DAMPs), activating the innate immune system response (Lu and Eguchi, 2020). Moreover, HSPs are key players in promoting proliferation, differentiation, invasion, and metastasis, which also gives cause for concern in case of their pharmacological upregulation (Calderwood and Murshid, 2017).

Hsp90, Hsp70, and Hsp27 content tends to elevate in tumors (Calderwood and Gong, 2016) increasing the risk of cancer (Dai et al., 2007; Garrido et al., 2006; Vanhooren et al., 2008). In this respect, stroke appears to be among the most appropriate diseases to which to apply chaperone-based interventions. Since there is only a narrow window in which to intervene with therapy, the risk of adverse effects is potentially lower than during long-term treatment; for example, in the context of neurodegenerative disorders.

In light of the latest discoveries, we can expect upcoming breakthroughs involving the application of chaperones in ischemic stroke treatment. In particular, the recently discovered family of Hero proteins possess features that make them excellent candidates for the therapy of stroke: low molecular weight, high stability, minimal immunogenicity, and energy-independent activity.

## 7 Limitations

- This work was based on the narrative literature review discussing biological insights and did not imply following the classical principles of meta-analysis or critical reviews such as assessing of statistical power and strict inclusion/exclusion criteria.
- The authors admit that the manuscript does not discuss the bulk part of the chaperome members: given the immense diversity of chaperones, only the most studied and classical subfamilies and members were reviewed. However, the potential contributions of other chaperone families that may also play significant roles.
- The actual pathogenesis of oxidative damage in stroke is constituted of myriads of links, and some of them were missing or not paid itemized description within the article. To generalize, the authors deliberately did not discuss multifaceted significance of oxidative damage in different stages of ischemic stroke, the roles of concomitant pathology etc.
- All theoretical justifications given in this article require experimental approval.
