# Supplementary material for: Chaperones vs. oxidative stress in the pathobiology of ischemic stroke
Source: Front Mol Neurosci. 2024 Dec 11;17:1513084. doi: 10.3389/fnmol.2024.1513084 (PMC11668803; doi:10.3389/fnmol.2024.1513084)
Supplement: Supplementary file 1 [file Table_1.docx]

Supplementary Material

Chaperones versus oxidative stress

in the pathobiology of ischemic stroke

Vladislav SOLDATOV, Artem VENEDIKTOV, Andrei BELYKH, Gennadii PIAVCHENKO, Mukhammad David NAIMZADA, Nastasya OGNEVA, Natalia KARTASHKINA, and Olga BUSHUEVA

***Corresponding Authors:** Vladislav Soldatov — [zinkfingers@gmail.com](mailto:zinkfingers@gmail.com), Olga Bushueva — [olga.bushueva@inbox.ru](mailto:olga.bushueva@inbox.ru).


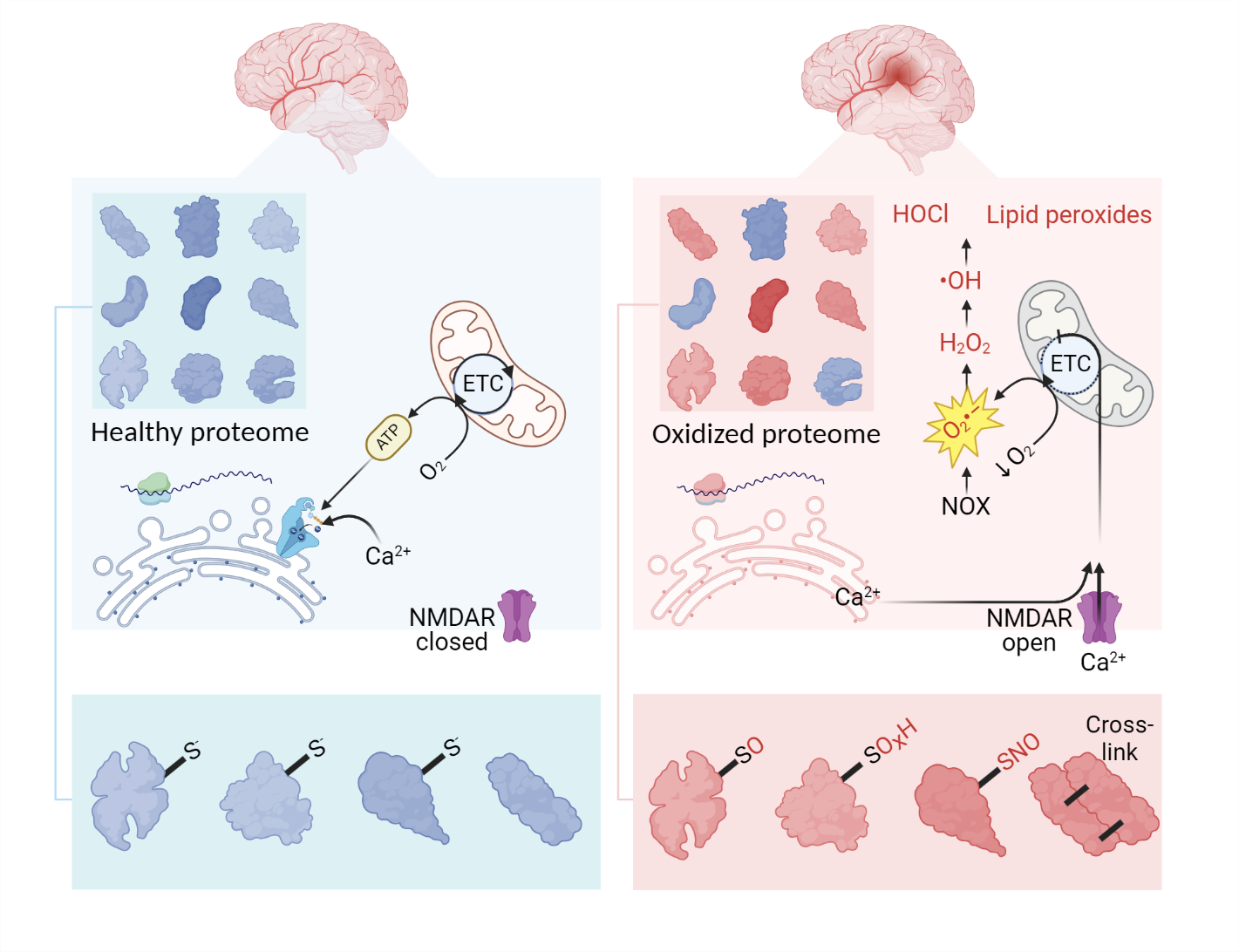


**Fig. 1. Outlines of oxidative stress and proteome overoxidation in ischemic stroke.** Normally, the healthy proteome is supported by balanced translation, modification, and utilization of proteins in homeostatic conditions. At the site of ischemia, a decrease in oxygen provokes mitochondria to release excessive superoxide launching the cascade of ROS production. This process is further enhanced by imbalanced Ca^2+^ as a result of ATP depletion, glutamate excitotoxicity, and increased levels of protons. Developing oxidative stress leads to overoxidation of the proteome, thus complicating the functioning of the proteins. Oxidative modifications of the proteome usually imply the addition of O, OH, O_2_H, O_3_H and NO groups to sulfur-containing amino residues. ETC – electron transport chain. Created in https://BioRender.com.


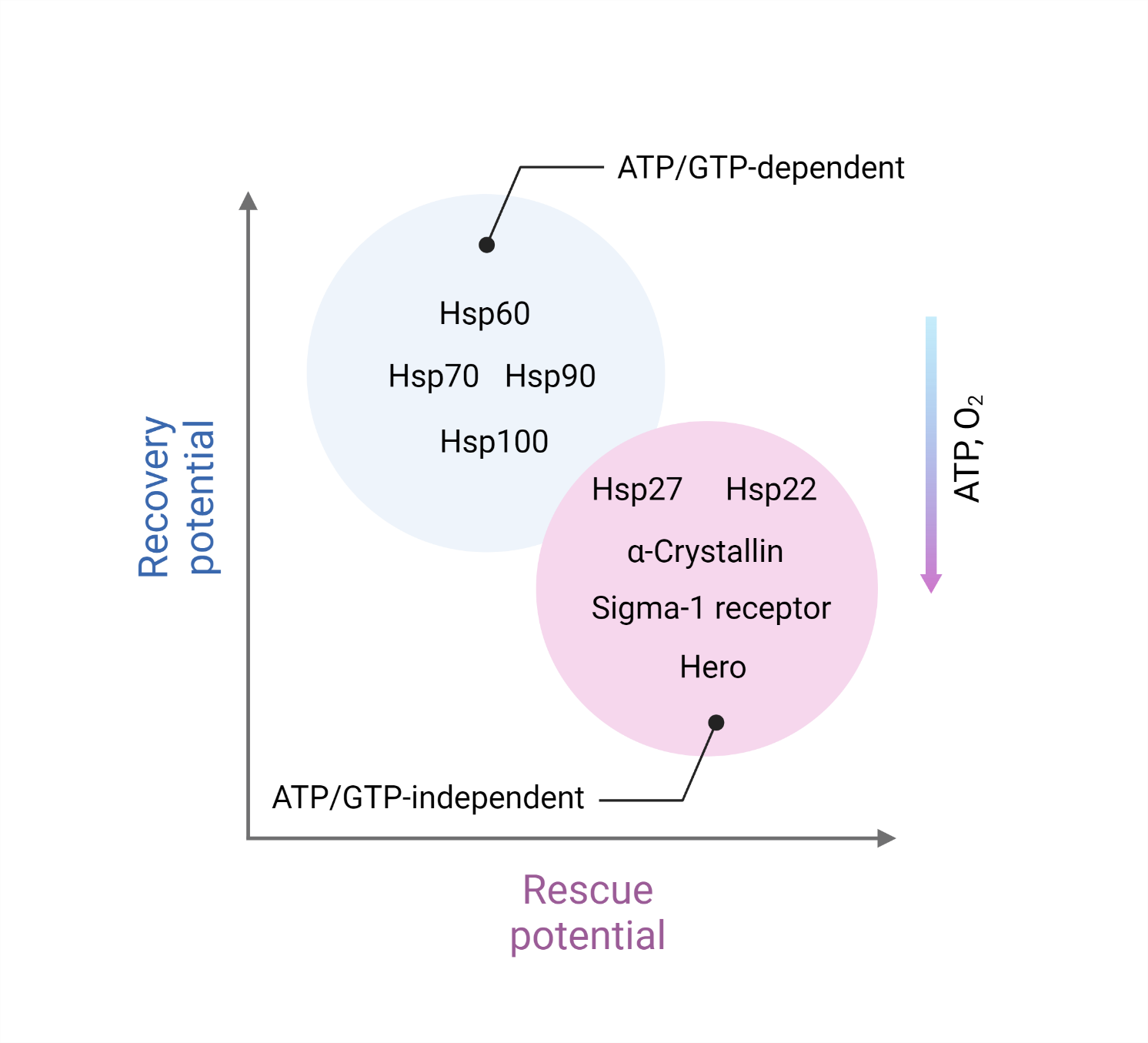


**Fig. 2.** **Distinct role of ATP-dependent and ATP-independent chaperones in ischemic stroke.** ATP-independent chaperones, including Hsp22, Hsp27, α-crystallin, Sigma-1 receptor and Hero, are especially vital in areas experiencing severe ATP depletion, closer to the ischemic core and in the acute phase of stroke. These chaperones function without the need for energy input from ATP, making them well-suited to stabilize and protect cellular proteins under energy-deficient conditions. They help prevent protein aggregation by binding to exposed hydrophobic regions of partially unfolded proteins, minimizing cellular damage when energy resources are critically low. On the other hand, ATP-dependent chaperones, which require ATP to assist in protein folding and repair, play a more prominent role in regions with moderate ATP depletion. These chaperones, such as Hsp60, Hsp70 and Hsp90 and Hsp100 are particularly important during the recovery phases following the initial stroke event. As ATP levels gradually restore, ATP-dependent chaperones can actively refold and repair damaged proteins, contributing to cellular recovery and enhancing resilience in the affected areas. This complementary activity of ATP-independent and ATP-dependent chaperones reflects a dynamic, phase-specific chaperone response aimed at protecting and restoring the proteome in different stages of stroke-induced cellular stress. Created in https://BioRender.com.

**Table 1. Key heat shock proteins (HSPs) in ischemic stroke.**

| Family/Member | | Functions | Localization in cell | Kinetics | References |
| --- | --- | --- | --- | --- | --- |
| Chaperonins / Hsp60 | | Folding and refolding of polypeptides in mitochondria  Posttranslational modifications of polypeptides in cytosol  Depends principally on co-chaperonin Hsp10  Activity is inhibited by nitration/hyperacetylation  Interacts with mortalin in mitochondria and cytoplasm | Predominantly mitochondria, less in Golgi, peroxisomes, plasma membrane, cytosol | ATP-dependent foldases | (Hartl, 1991; Soltys et al., 1997; Wadhwa et al., 2005; Caruso Bavisotto et al., 2020; Androvitsanea et al., 2021) |
| Hsp70 | Hsp70 / HspA1 | Folding of newly synthesized proteins  Anti-apoptotic activity in cytosol cascades  Hsp40-related proteostasis:  Ubiquitin-proteasome axis  CMA together with Hsp90  Micro- and macroautophagy  Stronger expression in stress reactions  Hsp40-dependent and counterpart of Hsp90 | Cytosol, lysosomes, plasma membrane, nucleus | ATP-dependent foldases  ATP-independent holdases* | (Flachbartová and Kovacech, 2013; Penna et al., 2018; Bilog et al., 2019; Androvitsanea et al., 2021) |
|  | Hsc70 | Constitutive member of Hsp70 family  Protection of nascent or unfolded proteins  Key regulator of physiological autophagy | Nucleus, less in cytosol and lysosomes | Strong ATP- and Hsp40-dependent foldase | (Rutledge et al., 2022) |
|  | Mortalin / mtHsp70 | Refolding of ROS-damaged proteins  Iron-sulfur cluster synthesis  Protein translocation to and from mitochondria  Interacts with Hsp60 via N-terminal region | Predominantly mitochondria, less in cytosol, | ATP-dependent foldase | (Wadhwa et al., 2005; Esfahanian et al., 2023) |
| Hsp90 | | Endoplasmic reticulum-located protein folding  CMA together with Hsp70  ATP-independent stress signaling  Common antagonism with Hsp70 | Either cytosolic, endoplasmic reticulum, or mitochondria-related forms, less in nucleus | ATP-dependent foldases  ATP-independent holdases* | (Jackson, 2012; Rutledge et al., 2022; Androvitsanea et al., 2021) |
| Small Hsp / Hsp20 and HspB8 | | Prevent aggregation of unfolded proteins  Recruit Hsp70 to selective autophagy | Nucleus, nucleolus, cytosol, mitochondria, endoplasmic reticulum | ATP-independent holdases | (Woehrer et al., 2015; Androvitsanea et al., 2021; Hu et al., 2022; Tedesco et al., 2023) |
| Hsp27 | | Protein quality control, referring to proteasome machinery, especially in oxidative stress  Holdase with strong anti-apoptotic activity  Prevents mitophagy  Co-enhances with Hsp70 | Cytosol and nucleus, often associated with cytoskeleton | ATP-independent holdase | (Stetler, 2009; Androvitsanea et al., 2021; Boyd, 2023) |

* – some chaperones are not strictly classified as holdases or foldases, possessing both types of activities.


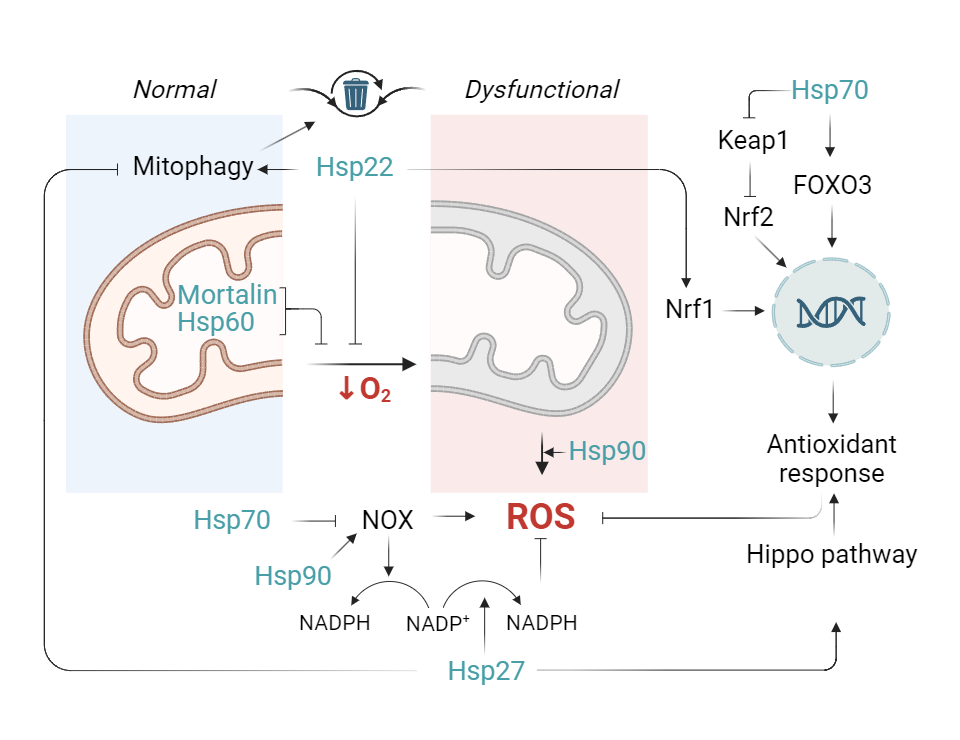


**Fig. 3. Outlines of the contribution of HSPs to controlling oxidative stress during ischemia.** Normal functioning of mitochondria implies moderate leakage of electrons and production of reactive oxygen species (ROS). However, facing a lack of oxygen, mitochondria produce excessive ROS and enter a dysfunctional state. In this scenario, Hsp22 limits mitochondrial activity, thus preventing ROS production. In parallel, mitochondrial Hsp70 (mortalin) and mitochondrial Hsp60 attenuate mitochondrial dysfunction via their chaperone-dependent protection of mitochondrial integrity and ETC. Hsp70 stimulates FOXO3 transcription factor activates Nuclear factor erythroid 2-related factor 2 (Nrf2) pathway and inhibits NOX activity while Hsp22 activates Nuclear factor erythroid 2-related factor 1 (Nrf1). Nrf1 and Nrf2 are both transcription factors known for their roles in regulating the expression of antioxidant and detoxifying enzymes Finally, Hsp27 is able to launch a cellular antioxidant response (such as an increase in superoxide dismutase and glutathione production) through Hippo pathway modulation (and probably other not yet revealed mechanisms), whereas Hsp90 appears to favor ROS production (partly through NOX enzymes activity upregulation). Created in https://BioRender.com.


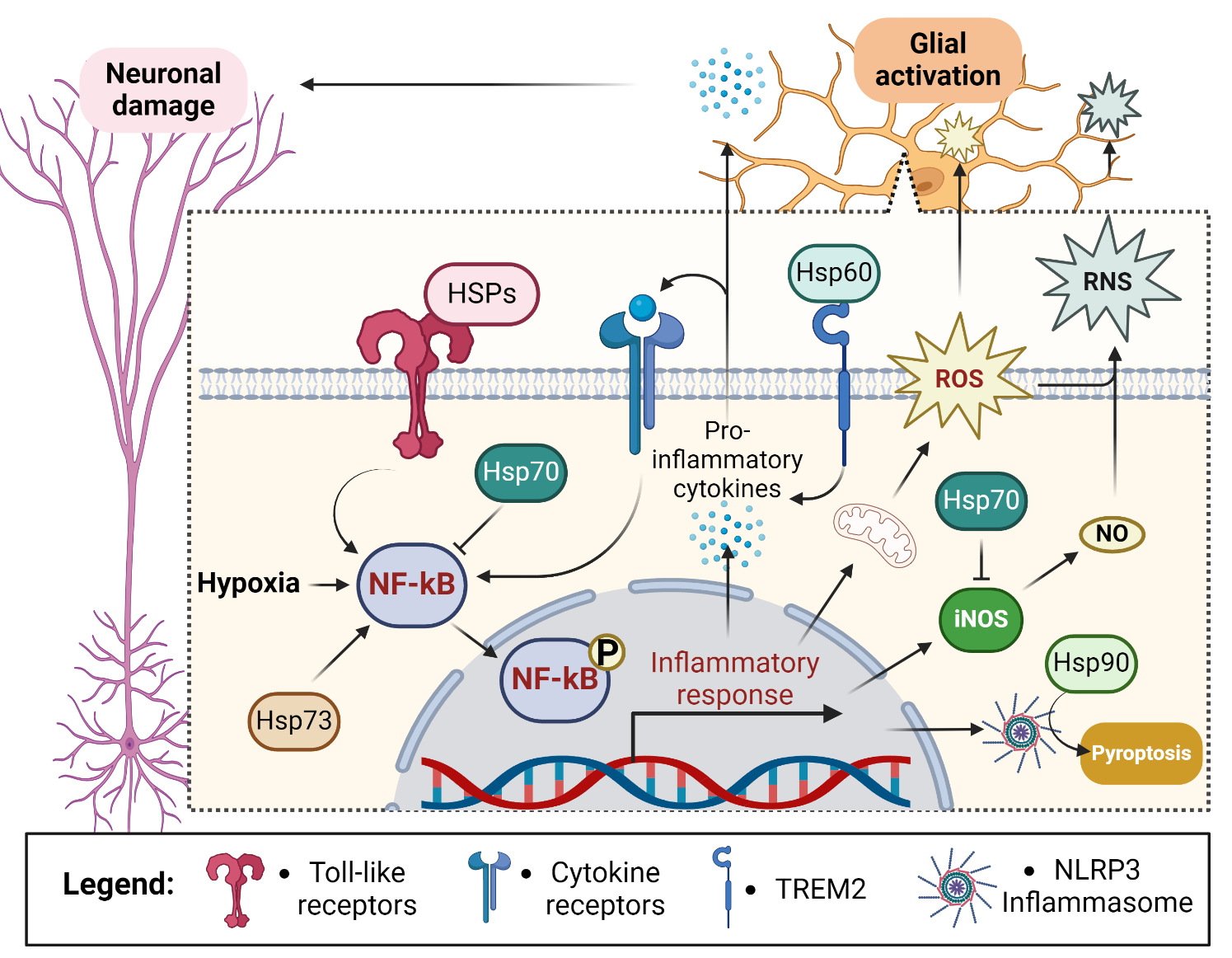


**Figure 4. Outline of chaperones’ contribution to the glial response during ischemic stroke.** Hypoxia stimulates neuroinflammation through the activation of NF-kB in glial cells. NF-kB leads to the assembly of NLRP3 inflammasome and secretion of pro-inflammatory cytokines sending inflammatory stimuli to neurons and glial cells themselves. Glial activation also exacerbates hypoxia-induced reactive oxygen species (ROS) production and leads to the generation of reactive nitrogen species (RNS) via inducible nitric oxide synthase (iNOS)-dependent release of nitric oxide (NO) (Zhu et al., 2022a). Altogether it leads to activation of pyroptosis and damage of proximal neurons. Intracellularly localized Hsp70 prevents activation of NF-kB and iNOS thus suppressing ROS and RNS generation. In contrast, extracellularly localized heat shock proteins (HSPs) activate NF-kB via toll-like receptor signaling and TREM2 (Hsp60). Additionally, intracellular Hsp73 activates NF-kB whereas intracellular Hsp90 stabilizes NLRP3 leading to inflammatory activation and pyroptosis. Created in https://BioRender.com.


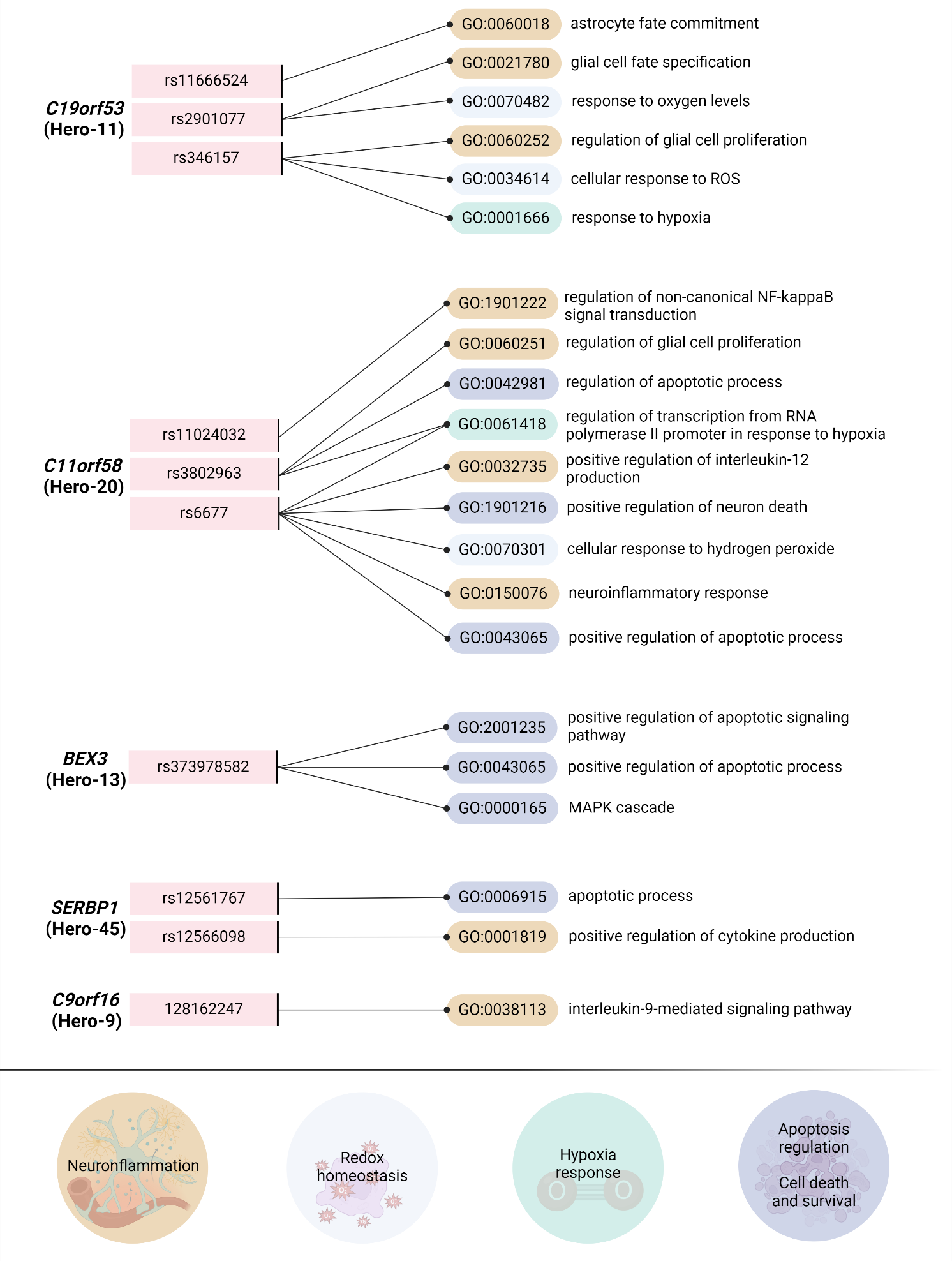


**Fig. 5. The analysis of overrepresented biological processes based on transcription factors associated with Hero-genetic polymorphisms.** Using the Gene Ontology online tool (http://geneontology.org/, accessed on October 23, 2024), we identified overrepresented biological processes directly related to the pathogenesis of ischemic stroke (IS) by analyzing the involvement of transcription factors associated with reference and SNP alleles. For this analysis, we focused on the most frequent single nucleotide polymorphisms (SNPs) or, when available, tagging SNPs (TagSNPs). From the list of biological processes linked to transcription factors associated with the reference and SNP alleles, we selected only those directly relevant to the pathobiology of ischemic stroke. As a result, we classified the biological processes into four groups: 'Neuroinflammation,' 'Redox Homeostasis,' 'Hypoxia Response,' and 'Apoptosis Regulation/Cell Death and Survival.' Importantly, we intentionally excluded processes related to neuronal proliferation, migration, and differentiation. The raw data for BEX3, C9orf16, and SERF2 are provided in the Supplementary File. Data for SERBP1, C11orf58 and C19orf53 are presented in Shilenok et al. (2023), Shilenok et al. (2024a), and Shilenok et al. (2024b), respectively. Created in https://BioRender.com.


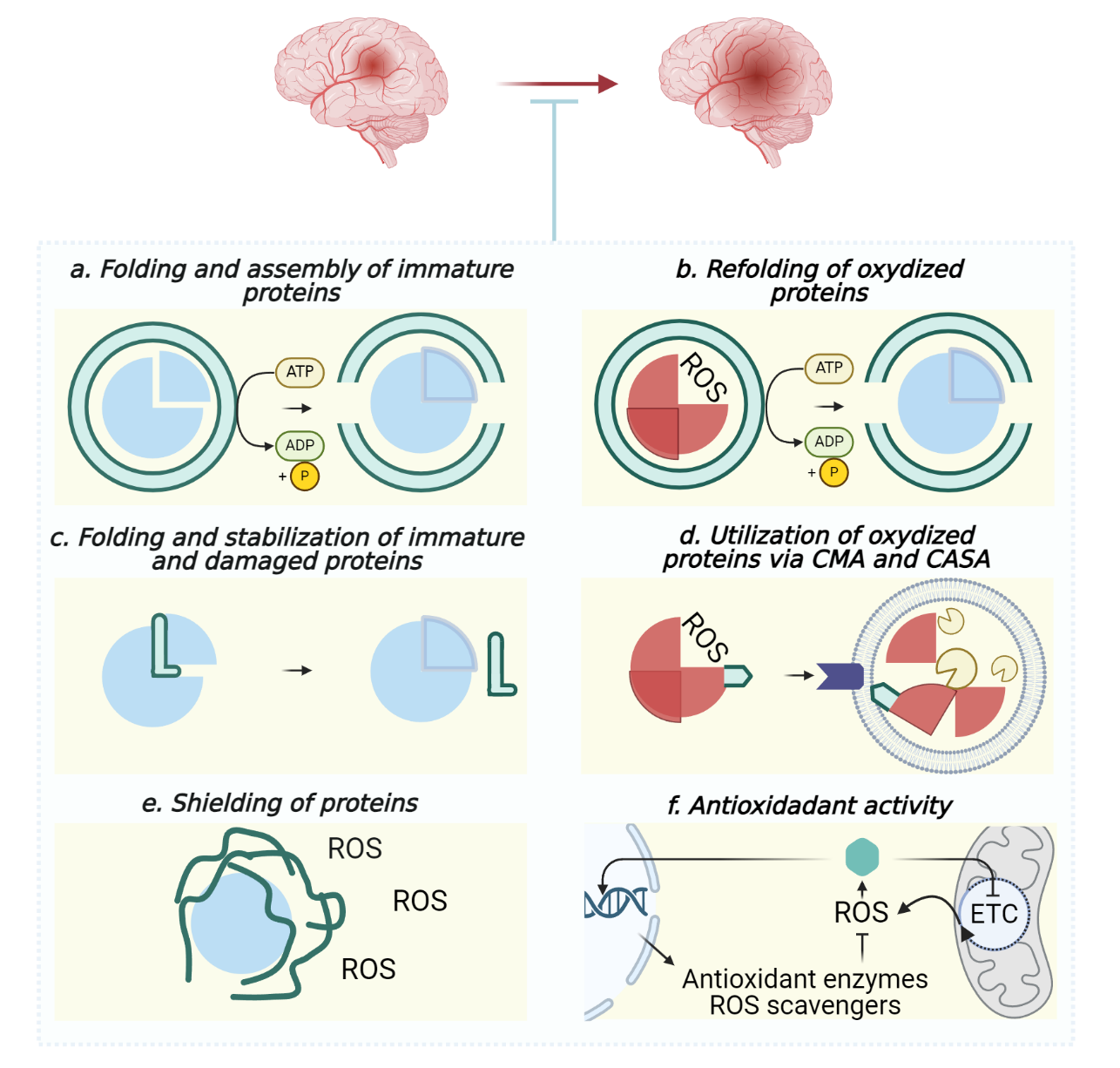


**Fig. 6. Outline of chaperones’ contribution to maintaining proteome quality during ischemic stroke.** In normal conditions (**a, c**) and to cope with overoxidation threats (**a–f**), chaperones co-interact with client proteins as foldases (**a, b**) or holdases (**c**), facilitate autophagic utilization of damaged polypeptides (**d**), shield client proteins to scavenge ROS (**e**) as well as provide ROS-sensing and activation of ROS-defense (**f**). Note: blue circles – undamaged proteins; red circles – damaged proteins; green objects interacting with blue or red circles – chaperones; round lipid envelope with yellow “pac-mans” inside – lysosome. **a, b** – Hsp60, Hsp70, Hsp90, Hsp100; **c** – Hsp22, Hsp27, α-Crystallin, Sigma-1 receptor, Hero-proteins; **d** – Hsc70 (CMA), Hsp70 and Hsp90 (CASA); **e** – Hero-proteins; **f** – Hsp22, Hsp27, Hsp70. Created in https://BioRender.com.
